# Dynamic system reliability under external shocks and internal markovian degradation

**DOI:** 10.1038/s41598-026-55143-2

**Published:** 2026-05-30

**Authors:** Vaibhav Bisht, Sunita Sharma, S. B. Singh

**Affiliations:** 1 Department of Mathematics, Jaypee University of Engineering and Technology, Guna, India; 2 Manipal Institute of Technology, Manipal Academy of Higher Education, Manipal, 576104 Karnataka India; 3 Department of Mathematics, Statistics and Computer Science, G. B. Pant University of Agriculture and Technology, Pantnagar, India

**Keywords:** $L_z$-transform, External Shock, Markovian Degradation, Reliability, and Availability, Engineering, Mathematics and computing

## Abstract

Systems operating in harsh environments face the dual challenge of external extreme shocks and internal degradation, a critical failure mode often overlooked in reliability engineering. This paper develops a novel $\:{L}_{z}$-transform model to analyze this coupled effect dynamically. Applied to a Doubly-Fed Induction Generator (DFIG), the model reveals a severe decline in performance: availability and reliability deteriorate asymptotically toward zero due to these combined stressors, with reliability falling more sharply due to the irreversibility of failures. To address this, a new maintenance strategy using dummy states is proposed. This approach dramatically improves system resilience, elevating both reliability and availability to near-perfect levels (~ 1.0). Comparative analysis shows the strategy completely mitigates time-dependent degradation, sustaining high performance indefinitely and improving vulnerable systems by orders of magnitude. Crucially, benefits are solely due to maintenance, not redundancy. This study proves proactive maintenance is indispensable for sustainable operation in shock-prone environments.

## Introduction

Reliability serves as a fundamental performance criterion for engineering systems, quantifying their ability to maintain intended functionality over time. In today’s world, where machines and engineered structures dominate nearly every aspect of life, reliability isn’t just a technical requirement; it’s a necessity for seamless operation, economic sustainability, and human safety. Whether it’s a mobile phone, a bridge, or a power grid, the demand for dependable performance means that reliability must be a top priority in design, operation and maintenance. Hence, the reliability arises as the key measure for assessing the performance of any engineering structure.

The reliability assessment of engineering structures, whether simple or complex, can be effectively conducted by modeling them as systems composed of interconnected subsystems arranged in specific configurations. Each subsystem, in turn, comprises various components or elements, which can demonstrate dichotomous or polymorphic behavior. This systematic approach enables a comprehensive performance evaluation, accounting for the interactions and dependencies between different elements within the overall structure. Early research on system reliability often assumed that components could exist in only two states: best or failed. Moreover, many real-world systems are not binary but instead function as multi-state systems (MSS). These systems can operate in multiple performance levels, varying from optimal functionality to absolute collapse, with varying levels of degradation in between. Moreover, the components of the system degrade from their best performing state either to intermediate states or failed state. This degradation may be caused either by internal degradation (like aging) or external shocks (abrupt change of mechanical stress, temperature, voltage, etc.). Aging induces progressive material degradation through mechanisms like fatigue and corrosion, reducing component reliability. Understanding aging effects is crucial for predicting remaining lifespan and implementing timely maintenance in critical infrastructure and industrial systems.

Moreover, the system may be affected by the external shocks simultaneously in addition to internal degradation^1,2^. An external shock refers to a sudden, unpredictable and potentially harmful event originating from outside a system or component that can cause total damage, degradation, or failure to the system^3^. In reliability engineering, shock models are widely used to analyze system failure mechanisms under external disturbances. These models can be categorized into four primary types: **(i)** the cumulative shock model, where failure occurs when the aggregate damage from successive shocks exceeds a critical threshold^4^; **(ii)** the extreme shock model, in which the system fails if any single shock event surpasses a predetermined severity level^5^; **(iii)** the $\:\delta\:$-shock model, where failure is triggered when the time interval between consecutive shocks falls below a specified duration $\:\delta\:$^6^; and **(iv)** the run shock model, which considers the system failed if a sequence of *n* consecutive shocks each exceed a critical magnitude^7^. Each model provides a distinct framework for evaluating reliability under different shock-induced failure scenarios. In addition to this, more than one shock model can be also combined to model the performance of systems^8–10^.

The area of research in reliability analysis has also evolved along with growing complex engineering systems, ranging from simple binary system to advanced multi-state system. Conventional techniques such as Fault Tree Analysis (FTA) and Reliability Block Diagrams (RBD) remain indispensable for binary systems, where components are classified discretely as either functional or failed. While these methods offer simplicity and clarity for straightforward scenarios, their inability to account for partial performance degradation or intermediate operational states limits their utility for MSS applications. To overcome these constraints, researchers have introduced more advanced analytical tools tailored for MSS. Among these, the Universal Generating Function (UGF), introduced by Ushakov^11^, stands out for its ability to consolidate diverse component states and their associated probabilities into a single, coherent arithmetic representation^12^.

Markov-based approaches are highly effective for modeling temporal reliability dynamics and state transitions, though their computational complexity grows substantially with larger systems. In contrast, Monte Carlo simulations offer exceptional flexibility for systems with complex dependencies and stochastic behaviors, but their high computational cost can be a limiting factor. A key challenge in MSS reliability analysis stems from the inherently dynamic nature of these systems, where component states evolve continuously over time. This temporal variability makes traditional static analysis methods insufficient for real-time reliability assessment. While the Universal Generating Function (UGF) technique has been widely adopted for MSS reliability evaluation^13–17^ and network analysis^18–20^, it is fundamentally limited by its dependence on random variables, confining its applicability to steady-state performance distributions. As a result, conventional UGF methods are unsuitable for analyzing transient phases, aging systems, or MSS subjected to time-varying stochastic loads. To address this limitation, the $\:{L}_{z}$-transform was introduced^21^ as an extension of the UGF framework, specifically tailored for discrete-state, continuous-time Markov processes. Unlike the classical UGF, which assumes static random variables and steady-state conditions, the $\:{L}_{z}$-transform captures the temporal evolution of system state probabilities, enabling reliability analysis of multi-state systems (MSS) under dynamic, time-varying environments. This advancement allows for the modeling of stochastic processes such as degradation, failure propagation, and state transitions over time. Consequently, the $\:{L}_{z}$-transform significantly broadens the scope of reliability assessment, providing a more accurate and flexible framework for representing real-world system behavior where time-dependent dynamics play a critical role. The $\:{L}_{z}$-transform has been extensively employed by researchers to assess the dependability of systems in real-time. For example, Frenkel et al.^22^ utilized this approach to evaluate the performance degradation of aging multi-state cooling systems in MRI machines. Lisnianski and Benhaim^23^ also used the $\:{L}_{z}$-transform to determine key reliability indicators for power stations. In another study, Frenkel et al.^24^ examined the operational stability of multi-source traction drives in Norilsk icebreaker vessels, assessing critical parameters such as availability, power generation, and deficits using the same technique. A comprehensive publication^25^ further explored this subject, presenting theoretical foundations, practical implementations, and case studies for dynamic reliability assessment of multi-state systems. Singh et al.^26^ advanced the methodology by developing an algorithm based on the $\:{L}_{z}$-transform to analyze the reliability of complex *m*-out-of-*r*-within-*k*-out-of-*n* systems with time-dependent component states. Additionally, Recent contributions in this field^27–32^ further underscore the significance of the $\:{L}_{z}$-transform in advance reliability engineering.

Various models have been developed by researchers to study the effect of shocks on system performance. Wang et al.^33^ developed the new competing risks model for systems suffering from a three-stage natural degradation process and random shocks. Wang et al.^34^ provided a novel self healing mechanism wherein the multi-state system damaged by prior harmful shocks can undergo stochastic recovery. D’Amico and Petroni^35^ studied the failure, repair and mobility Rate of Wind-Farm using the Markov process. Jiang and Jia^36^ developed the Markov renewal process to evaluate the reliability under continuous fatigue degradation and shock processes. The $\:{L}_{z}$-transform method offers significant computational advantages over the straightforward Markov approach for multi-state systems. While a direct Markov method requires solving $\:\prod\:_{j=1}^{n}{k}_{j}$ differential equations, leading to exponential state space growth, the $\:{L}_{z}$-transform reduces this to only $\:\sum\:_{i=1}^{n}{k}_{i}\:$equations by modeling each component separately. This drastically decreases computational complexity, avoids the “dimensionality curse,” and provides a formalized, error-resistant procedure for analyzing complex multi-state systems. Despite the extensive uses of the $\:{L}_{z}$-transform in system engineering, existing studies have not yet addressed its use in shock modeling. This paper bridges this gap by extending the conventional $\:{L}_{z}$-transform methodology to evaluate the dynamic reliability of systems subjected to combined shock modeling and internal Markov degradation. Furthermore, since maintenance strategies significantly influence system performance, we introduce a novel $\:{L}_{z}$-transform based maintenance strategy to enhance system reliability measures. The following table clearly distinguishes this study from existing research as:

**Table 1:** Distinction of the proposed study from existing research.

Feature	Existing $\:{\boldsymbol{L}}_{\boldsymbol{z}}$-transform studies [21–31]	Existing shock-degradation studies [4–10]	This Study
*Uses* $\:{L}_{z}$*-transform*	✓	×	✓
*Models Internal Markov Degradation*	✓	✓/×	✓
*Models External Shocks*	×	✓	✓
*Embeds shocks into* $\:{L}_{z}$*-transform*	×	×	✓
*Dummy state Maintenance*	×	×	✓

This work seeks to accomplish the following:

- To develop a system model based on internal degradation and evaluate its impact on dynamic system reliability indices using the $\:{L}_{z}$-transform. The internal degradation follows a Markov process, where the transition probability to the next state depends solely on the current state, independent of prior states.
- To extend the model by incorporating extreme external shocks alongside internal Markov degradation and assess how their combined influence affects system reliability measures.
- To introduce a novel maintenance strategy addressing both internal degradation and the combined effect of shocks and degradation. The effectiveness of this strategy will be evaluated by comparing its results with baseline reliability metrics.
- To analyze the impact of shock tolerance thresholds on system availability under both maintained and non-maintained conditions for specified performance levels.

## $\:{\boldsymbol{L}}_{\boldsymbol{z}}$-transform

A Discrete-State Continuous-Time (DSCT) Markov process can be defined as $\:G\left(t\right)=\{S,$ A, $\:{p}_{0}$}, where:

- * S* denotes the finite set of possible states, such that $\:G\left(t\right)\in\:\{\:{g}_{1},\:{g}_{2,\dots\:\dots\:\dots\:,}{g}_{M}\}$.
- $\:A\left(t\right)=\left[{a}_{ij}\left(t\right)\right]$ (for $\:i,j=\mathrm{1,2},\dots\:,M$) is the time-dependent transition rate matrix.
- $\:{p}_{0}$ represents the initial state probability distribution, expressed as:

$$\:{p}_{0}=[\mathrm{P}\left\{G\left(0\right)={g}_{1}\right\},\:\mathrm{P}\left\{G\left(0\right)={g}_{2}\right\}\dots\:\dots\:\dots\:\dots\:..,\mathrm{P}\left\{G\left(0\right)={g}_{M}\right\}]$$

The $\:{L}_{z}$-transform for such a process$\:\:G\left(t\right)$ is formulated as:

1 $$\:L_{z} \left( {G\left( t \right)} \right) = \:f\left( {z,\:t,\:p_{0} } \right) = \:\sum {\:_{{j = 1}}^{M} } p_{{ij}} \left( t \right)z^{{g_{{ij}} }}$$

where $\:{p}_{ij}$ denotes the transition probability from state * i* to $\:j\:$at time * t*, and * z* is a complex variable. It can be observed that the formula of both UGF and $\:{L}_{z}$-transform look similar but they are not same, the $\:{L}_{z}$-transform is not UGF, rather it is a new mathematical object as the $\:{L}_{z}$-transform essentially depends on time *t* and on initial conditions of Markov process $\:{p}_{0}$.

### Operational Rule for $\:{\boldsymbol{L}}_{\boldsymbol{z}}$-transform

The UGF (Universal Generating Function) operator $\:{\mathrm{Ω}}_{f}$ can be applied to the $\:{L}_{z}$*-*transforms of two independent Markov processes, $\:M\left(t\right)$ and $\:N\left(t\right)$, to derive the $\:{L}_{z}$*-*transform of their functional combination $\:f\left(M\right(t),\:N(t\left)\right)$. Mathematically.

2 $$L_{z} \left\{ {f(M\left( t \right),~N\left( t \right)} \right\} = {{\Omega }}_{f} \left( {L_{z} \left\{ {M\left( t \right)} \right\},~L_{z} \left\{ {N\left( t \right)} \right\}} \right)$$

### $\:{\boldsymbol{L}}_{\boldsymbol{z}}$-transform combined to shock

To embed the shock tolerance threshold into the reliability model, the traditional $\:{L}_{z}$-transform is extended by incorporating a shock-related parameter. The modified transform is defined as.

$$\:{L}_{z}\left(G\left(t\right)\right)\:=\sum\:_{j=1}^{M}{p}_{ij}\left(t\right){z}^{{\{g}_{ij},{\:{\delta\:}}_{ij}\}\:\:}$$

where $\:{p}_{ij}$ is the transition probability from state * i* to state * j* at time * t*, * z* is a complex variable, and $\:{\:{\delta\:}}_{ij}$ represents the maximum shock magnitude tolerated by the component * i* at state * j*. This term embeds the shock tolerance threshold into the transform, enabling the modeling of failure behavior under external shock impacts.

### The proposed shock model

The proposed model is specifically designed for multi-state, repairable, shock-sensitive systems characterized by discrete performance levels. Such systems are common in engineering applications where components exhibit distinct operational states (e.g., normal, derated, failed) and are subject to both internal degradation mechanisms (e.g., fatigue, wear, corrosion) and external random shocks (e.g., grid disturbances, voltage sags, mechanical vibrations, environmental loads). Typical application domains include wind turbine generators, doubly-fed induction generator (DFIG) systems, power electronic converters, and energy storage systems. A system is composed of various constituent elements arranged in a specific topological configuration. Typically, each component can exist in multiple states; three, four, or more depending on the system’s complexity. Such components are referred to as Multi-State Elements (MEs), and the system itself is termed a Multi-State System (MSS). The states of an ME are not static; rather, they evolve over time due to internal degradation, transitioning gradually from the highest performance state to intermediate states and, eventually, to complete failure.

The system operates under external environmental conditions and is subject to random external shocks, which may occur at any state alongside continuous internal degradation. Each ME possesses a shock absorption capacity, which varies with its performance state. Higher-performing states exhibit greater resilience to shocks, whereas lower-performing states are more susceptible to failure. Thus, the maximum bearable shock differs across states. Let us consider an MSS comprising multiple MEs, where each ME has * M* distinct states, ordered from 1 (complete failure) to * M* (optimal performance), with intermediate states * M-i* (where $\:1\:<\:i\:<\:M$) representing progressively degraded performance levels. This study focuses on modeling the impact of extreme shocks on system reliability. An extreme shock, denoted by $\:\delta\:$, causes failure of the system if its magnitude exceeds a predefined threshold. When an extreme shock strikes an ME in state * K* (where $\:1\:<\:K\:\le\:\:M$), two scenarios arise:

a) **Shock Absorption**: When an extreme shock $\:\delta\:$ strikes a ME in state * K*, and its intensity is less than the tolerable limit of state * K* ($\:\delta\:\:\le\:{\delta\:}_{K}$), the system exhibits a controlled degradation response. The ME transitions from state * K* to the next lower performance state (* K-1*) at a minor failure rate $\:{\lambda\:}_{K,\:K-1}$, reflecting gradual wear rather than sudden failure. In other simpler words, when the shock intensity of extreme shock is within the tolerable limit ($\:\delta\:\:\le\:{\delta\:}_{K}$), the component degrades gradually to the next lower performance state at a minor failure. Here $\:\delta\:$ denotes the intensity of the shock event, and $\:{\delta\:}_{K}$ represents the maximum shock tolerance threshold of the component when operating in state * K*. In this process, the shock is absorbed by the state * K*. This mechanism allows the system to remain operational, albeit with reduced efficiency. For example, a power generator in state 4 subjected to a manageable electrical surge may degrade to state 3 at rate $\:{\lambda\:}_{43}$, sustaining operation but with diminished output. Such minor failures accumulate over time, necessitating maintenance to prevent progressive performance decline.

b) **Shock-Induced Failure**: If the shock intensity exceeds the ME’s capacity ($\:\delta\:>{\delta\:}_{K}$), catastrophic failure occurs, bypassing intermediate states and transitioning directly to complete failure (state 1). In other words, when the shock intensity ($\:\delta\:$) exceeds the maximum shock tolerance threshold of state * K* ($\:{\delta\:}_{K}$) i.e., $\:\delta\:>{\delta\:}_{K}$, the component fails catastrophically, transitioning directly to the complete failure state. This type of failure is designated as the shock-induced failure since they are caused by external extreme shock. This abrupt failure follows an exponential rate $\:{e}^{{\lambda\:}_{K1}t}$., where $\:{\lambda\:}_{K1}$ captures the element’s vulnerability to extreme shocks. The model accounts for both instantaneous failure risk and time-dependent damage accumulation. For instance, a generator hit by a severe lightning strike ($\:\delta\:>{\delta\:}_{4}$) may suffer irreparable damage, failing immediately at rate $\:{e}^{{\lambda\:}_{41}t}$. Such failures are critical in high-risk systems (e.g., aerospace, nuclear plants), where they can trigger cascading breakdowns, underscoring the need for robust safety margins and rapid response protocols.

**Fig. 1:**
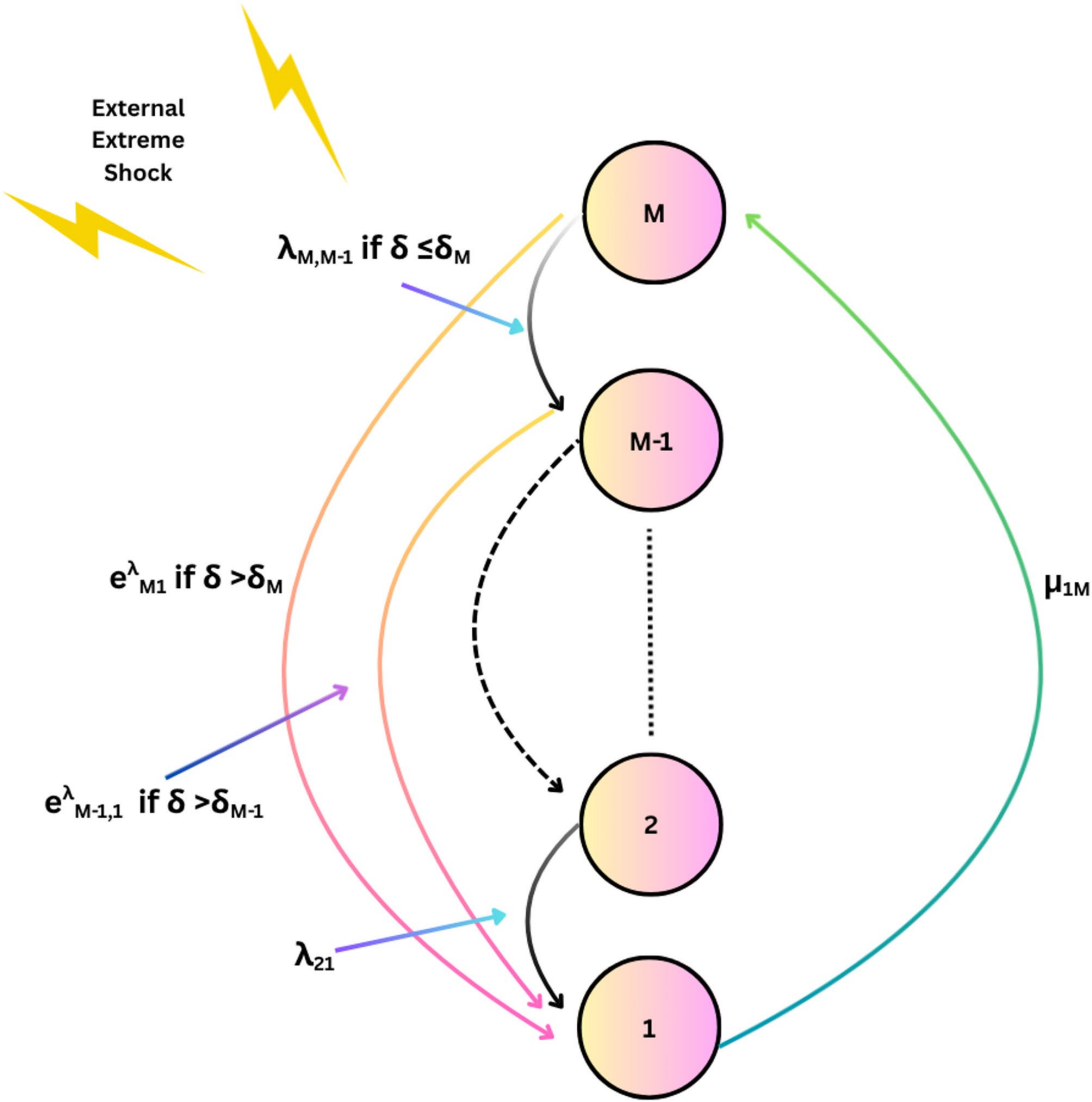
State transition diagram.

The state transition diagram of any ME for the proposed shock model is shown in Fig. 1:

### The proposed maintenance strategy

In reliability theory, maintenance plays a crucial role in preventing failures and restoring systems to ensure sustained performance and operational availability. Effective maintenance strategies are essential for enhancing system longevity, reliability, and availability. In Sect.  3, we modeled the simultaneous impact of external shocks and internal degradation and derived dynamic reliability measures using the $\:{L}_{z}$*-*transform. Building on this foundation, this section introduces a novel maintenance strategy that addresses combined extreme external shocks and internal Markov degradation.

**Fig. 2:**
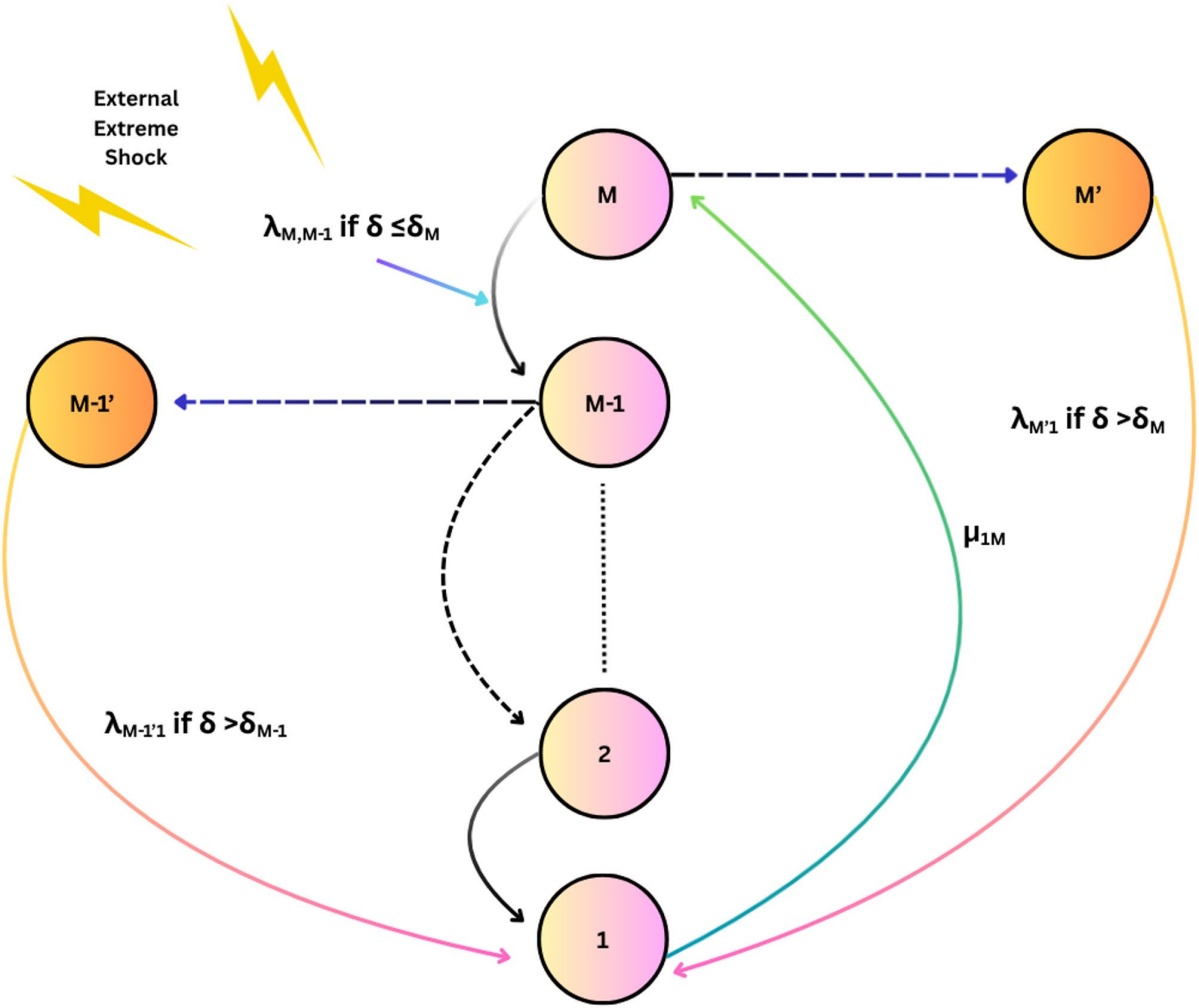
State transition diagram as per maintenance.

As previously discussed, systems are susceptible to external shocks, which can occur at any operational state. The proposed maintenance strategy focuses on restoring states affected by extreme external shocks. Consider a MSS where a shock impacts state * K* of a multi-state element (ME). Instead of transitioning to state * K-1* or state 1 (based on the system’s shock tolerance limit), the system moves to a standby dummy state $\:K{\prime\:}$. The dummy state is a state where the component does not operate normally but maintains the same performance level as the preceding operational state. This dummy state $\:K{\prime\:}$ serves as an active backup, immediately taking over when a shock disrupts state * K*. It retains the same performance level and shock tolerance as state * K*, effectively resetting the shock’s impact. From $\:K{\prime\:}$, internal degradation continues as before, but the transition to the major failure state (state 1) occurs with a reduced transition intensity $\:{\lambda\:}_{{K}^{{\prime\:}}1}$, rather than the original $\:{e}^{{\lambda\:}_{K1}t}$, since the dummy state mitigates the shock’s effect. All other repair mechanisms remain unchanged: repairs are only performed when the system reaches the state 1, after which it returns back to the best state, * M* with transition intensity $\:{\mu\:}_{1M}$. The motivation corresponding to this maintenance policy is to optimize system availability under the combined influence of external shocks and internal degradation. The state transition diagram according to the proposed maintenance strategy is shown in Fig. 2.

### Real life case study: wind turbine generator

**Fig. 3:**
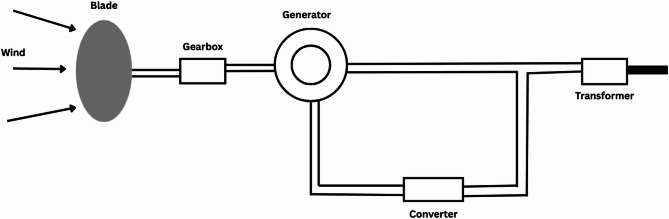
Doubly-fed induction generator (DFIG)^37^.

Wind turbine generators serve as the indispensable electromechanical core that harnesses aerodynamic forces to generate electrical current^37^. Based on their output capacity, these generators are classified into small, medium, large, and megawatt-scale systems. Among these, the Doubly-Fed Induction Generator (DFIG) has emerged as a dominant technology in the wind power market due to its operational stability and high efficiency. A DFIG, as shown in Fig. 3, consists of five primary components:

• **Blade**: Captures wind energy, generating torque that initiates mechanical rotation.

• **Gearbox**: Increases the rotational speed of the shaft before transferring mechanical energy to the generator.

• **Generator**: Converts mechanical energy into electrical energy.

• **Converter**: Excites the rotor and ensures the stator output voltage matches the grid in amplitude, frequency, and phase. Without the converter, the generator cannot function properly.

• **Transformer**: Adjusts voltage levels for grid integration.

**Fig. 4:**
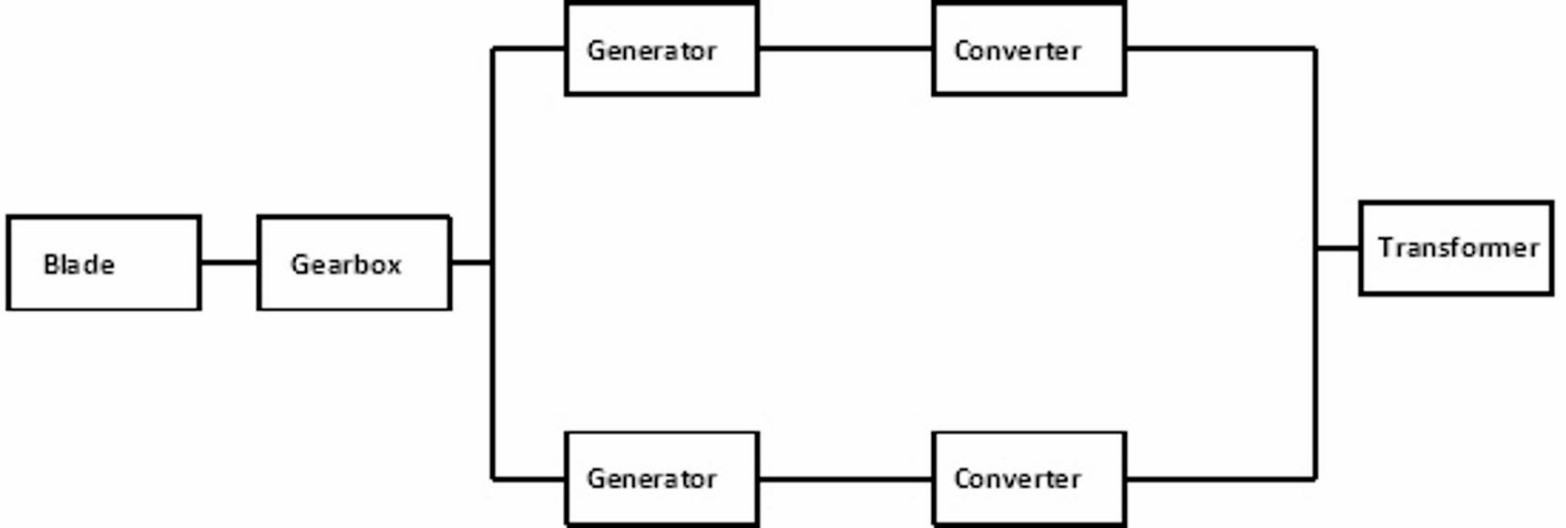
Reliability block diagram of a DFIG.

The operational sequence begins when wind rotates the blades, producing mechanical torque. This torque is amplified by the gearbox and transferred to the generator, where it is converted into electrical energy. The converter plays a critical role in regulating rotor excitation, ensuring stable power output. The reliability block diagram of a DFIG (Fig. 4) illustrates its redundant design, featuring two parallel systems, each comprising a generator and converter, connected in series with the blade, gearbox, and transformer. Each component within the DFIG operates across multiple performance states, as detailed in Table 2. The blade and gearbox, being mechanical elements, exhibit four distinct states corresponding to progressive wear levels, ranging from optimal function to near-failure conditions. In contrast, electrical components, the generator, converter, and transformer transition through three states influenced primarily by the degradation of IGBT modules, which compromises performance even if full functionality persists temporarily.

The gradual degradation in operational efficiency classifies the DFIG as a MSS, where components experience progressive performance deterioration rather than an abrupt transition from functional to failed states. These components are subjected to both external shocks and internal wear over time, with Table 2 defining the maximum shock tolerance for each component at different degradation states. This study aims to develop a unified model that accounts for the simultaneous impact of extreme external shocks and internal degradation, as detailed in Sect.  3, while evaluating how maintenance strategies influence the overall performance of wind turbine DFIG systems. The analysis will be conducted in two phases with the help of $\:{L}_{z}$*-*transform:

• **Combined Shock and Degradation Analysis**: Assessing the joint effects of external shocks and internal wear on dynamic reliability and availability, along with the proposed maintenance strategy.

• **Performance and Availability Evaluation**: Investigating how varying shock tolerance thresholds affect system availability, both with and without maintenance, for specified performance requirements.

By integrating these factors, the study seeks to optimize maintenance approaches and enhance the performance of DFIG-based wind power systems.

**Table 2:** DFIG’s transition and performance rates.

Wind Turbine Generator		Transition Rates of Components				Performance Level			Transition Rates of Components			Performance Level
	States	S_1_	S_2_	S_3_	S_4_	$\:{\boldsymbol{g}}_{\boldsymbol{i}\boldsymbol{j}}$		States	S_1_	S_2_	S_3_	$\:{\boldsymbol{g}}_{\boldsymbol{i}\boldsymbol{j}}$
	S_1_	0.0	0.0	0.0	0.6	0		S_1_	0.0	0.0	0.5	0
1) Blade	S_2_	0.00001	0.0	0.0	0.0	15	3)Generator	S_2_	0.00012	0.0	0.0	20
	S_3_	0.0000028	0.00004	0.0	0.0	30		S_3_	0.000010	0.00025	0.0	40
	S_4_	0.000005	0.0	0.00007	0.0	50						
	S_1_	0.0	0.0	0.0	0.4	0		S_1_	0.0	0.0	0.3	0
2) Gearbox	S_2_	0.00002	0.0	0.0	0.0	20	4)Convertor	S_2_	0.00009	0.0	0.0	23
	S_3_	0.0000046	0.00006	0.0	0.0	34		S_3_	0.000007	0.00018	0.0	52
	S_4_	0.000008	0.0	0.00012	0.0	48						
	S_1_	0.0	0.0	0.7	-	0						
5)Transformer	S_2_	0.00008	0.0	0.0	-	28						
	S_3_	0.000006	0.00015	0.0	-	55						

**Table 3:** Maximum tolerable shock.

Component	State 4	State 3	State 2	State 1
Blade	2.2	1.7	0.8	0
Gearbox	2.6	1.9	1.1	0
Generator	-	3.0	2.0	0
Convertor	-	2.8	1.7	0
Transformer	-	2.5	0.7	0

### Steps to obtain the dynamic reliability measures of the DFIG system in accordance to the proposed model with the help of $\:{\boldsymbol{L}}_{\boldsymbol{z}}$*-*transform

As established in the previous section, the Doubly-Fed Induction Generator (DFIG) comprises five major electromechanical components: the Blade, Gearbox, Generator, Converter, and Transformer. The Blade and Gearbox each operate across four distinct performance states, while the Generator, Converter, and Transformer each function within three states. These states are numerically designated, with State 4 (for Blade and Gearbox) or State 3 (for other components) representing optimal performance, and State 1 consistently indicating complete failure. The intermediate states (State 2 and State 3 for mechanical components; State 2 for electrical components) reflect progressively degrading performance levels. The specific transition rates between these states for each component are detailed in Table 2.

The most effective single method to derive the $\:{L}_{z}$*-*transform involves first constructing the system of differential equations that define the state probabilities, as formalized in Eq. (3). These equations capture the time-dependent evolution of state probabilities based on Markovian transition rates between states in a multi-state system.

3 $$\begin{aligned}\frac{dp_{i1}(t)}{dt}&=\sum_{e=2}^{M_i} \lambda_{e1}^{(i)} p_{ie}(t)-p_{i1}(t)\sum_{e=2}^{M_i} \mu_{ie}^{(i)}\\\frac{dp_{ij}(t)}{dt}&=\sum_{e=j+1}^{M_i} \lambda_{ej}^{(i)} p_{ie}(t)+\sum_{e=1}^{j-1} \mu_{ej}^{(i)} p_{ie}(t)-p_{ij}(t)\left(\sum_{e=1}^{j-1} \lambda_{je}^{(i)}+\sum_{e=1}^{M_i} \mu_{je}^{(i)}\right)\\\frac{dp_{iM_i}(t)}{dt}&=\sum_{e=1}^{M_i-1} \mu_{eM_i}^{(i)} p_{ie}(t)-p_{iM_i}(t)\sum_{e=1}^{M_i-1} \lambda_{M_ie}^{(i)}\end{aligned}$$

For the first component (* i=1*, Blade), we examine its behavior when subjected to external extreme shocks while operating in States 4 and 3. As established in Sect.  3, the component’s response depends on whether the shock intensity exceeds its maximum tolerable threshold. When the shock intensity remains below this critical limit, the Blade undergoes gradual degradation: transitioning from State 4 to State 3 with rate $\:{\lambda\:}_{43}$, and from State 3 to State 2 with rate $\:{\lambda\:}_{32}$. However, when shock intensity surpasses the component’s tolerance, catastrophic failure occurs immediately, with State 4 failing to State 1 at a time-dependent rate of $\:{e}^{{\lambda\:}_{41}t}\:$and State 3 failing to State 1 at rate $\:{e}^{{\lambda\:}_{31}t}$. These transition dynamics, when incorporated into the system of differential equations using the parameters from Table 2, yield the modified formulation for our proposed model as shown in the subsequent equations.

4 $$\begin{gathered} \frac{{dp_{{11}} \left( t \right)}}{{dt}} = - 0.6.p_{{11}} \left( t \right) + 0.00001.p_{{12}} \left( t \right) + e^{{0.0000028t}} .p_{{13}} \left( t \right) + e^{{0.000005t}} .p_{{14}} \left( t \right) \hfill \\ \frac{{dp_{{12}} \left( t \right)}}{{dt}} = - 0.00001.p_{{12}} \left( t \right) + 0.00004.p_{{13}} \left( t \right) \hfill \\ \frac{{dp_{{13}} \left( t \right)}}{{dt}} = - e^{{\left( {0.0000028t~ + ~0.00004} \right)}} p_{{13}} \left( t \right) + 0.00007.p_{{14}} \left( t \right) \hfill \\ \frac{{dp_{{14}} \left( t \right)}}{{dt}} = 0.6.p_{{11}} \left( t \right) - e^{{\left( {0.000005t~ + ~0.00007} \right)}} .p_{{14}} \left( t \right) \hfill \\ \end{gathered}$$

**Fig. 5:**
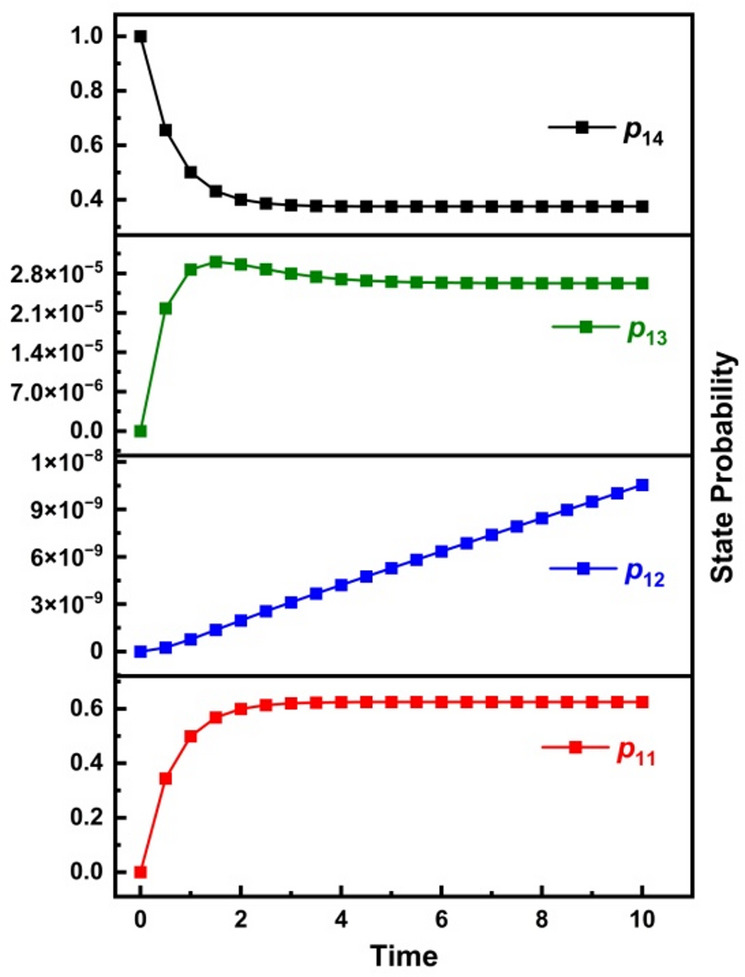
State probabilities of the blade.

Now these system of differential equations are solved under initial conditions $\:{p}_{11}\left(0\right)={p}_{12}\left(0\right)={p}_{13}\left(0\right)=0$ and $\:{p}_{14}\left(0\right)=1$ to obtain the transient state probabilities as depicted in Fig. 5.

Having established the transient state probabilities for the initial component (the Blade), its corresponding $\:{L}_{z}$*-*transform can be subsequently refined and recalibrated for the proposed model by incorporating maximum tolerable shock in the traditional $\:{L}_{z}$*-*transform as given in Eq. (1) and also substituting the values from Tables 2 and 3 as:

$$\begin{gathered} L_{z} \left( {G_{1} \left( t \right)} \right)\mathop \sum \limits_{{j = 1}}^{4} p_{{1j}} \left( t \right)z^{{\{ g_{{1j}} ,~{\delta }_{{1j}} \} ~~}} \hfill \\ = ~p_{{11}} \left( t \right)z^{{\left\{ {g_{{11}} ,~{\delta }_{{11}} } \right\}}} + p_{{12}} \left( t \right)z^{{\left\{ {g_{{12}} ,~{\delta }_{{12}} } \right\}}} + p_{{13}} \left( t \right)z^{{\left\{ {g_{{13}} ,~{\delta }_{{13}} } \right\}}} + ~p_{{14}} \left( t \right)z^{{\left\{ {g_{{14}} ,~{\delta }_{{14}} } \right\}}} \hfill \\ = ~p_{{11}} \left( t \right)z^{{\left\{ {0,~~0} \right\}}} + p_{{12}} \left( t \right)z^{{\left\{ {15,~~0.8} \right\}}} + p_{{13}} \left( t \right)z^{{\left\{ {30,~~1.7} \right\}}} + p_{{14}} \left( t \right)z^{{\left\{ {50,~~2.2} \right\}}} \hfill \\ \end{gathered}$$

Similarly, for the proposed shock model, the system of differential equation for the second component (Gearbox) can be formed with the help of Table 2 as:

5 $$\begin{gathered} \,\frac{{dp_{{21}} \left( t \right)}}{{dt}} = - 0.4.p_{{21}} \left( t \right) + 0.00002.p_{{22}} \left( t \right) + e^{{0.0000046t}} .p_{{23}} \left( t \right) + e^{{0.000008t}} .p_{{24}} \left( t \right) \hfill \\ \frac{{dp_{{22}} \left( t \right)}}{{dt}} = - 0.00002.p_{{22}} \left( t \right) + 0.00006.p_{{23}} \left( t \right) \hfill \\ \frac{{dp_{{23}} \left( t \right)}}{{dt}} = - e^{{\left( {0.0000046t~ + ~0.00006} \right)}} p_{{23}} \left( t \right) + 0.00012.p_{{24}} \left( t \right) \hfill \\ \frac{{dp_{{24}} \left( t \right)}}{{dt}} = 0.4.p_{{21}} \left( t \right) - e^{{\left( {0.000008t~ + ~0.00012} \right)}} .p_{{24}} \left( t \right) \hfill \\ \end{gathered}$$

**Fig. 6:**
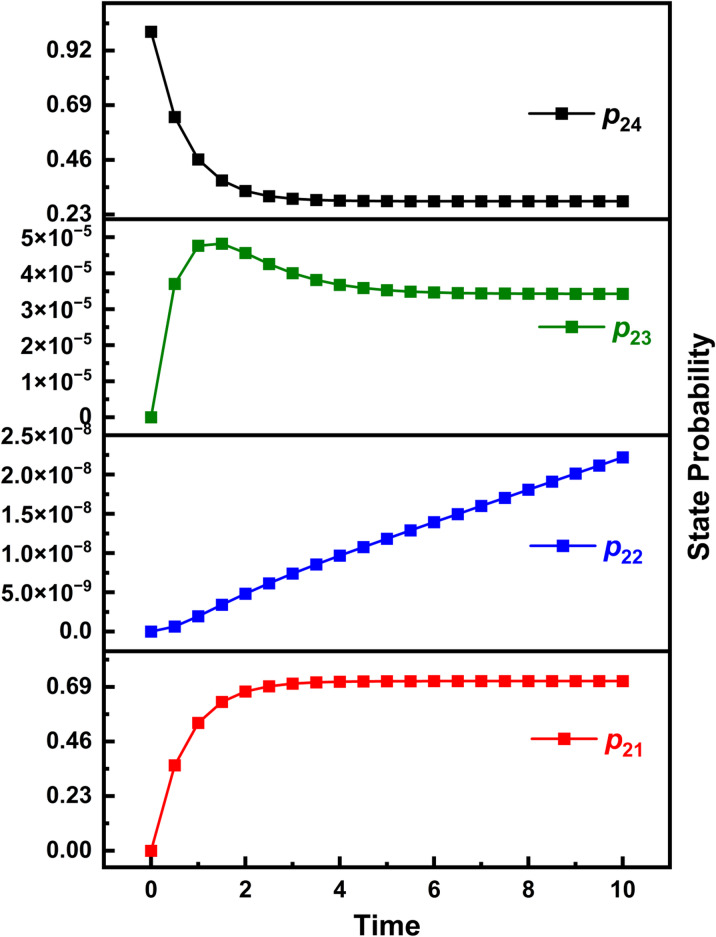
Transient probabilities of the gearbox.

The state probabilities for the system, visualized in Fig. 6, were derived by numerically solving the system of differential Eq. (5) under the initial conditions $\:{p}_{21}\left(0\right)={p}_{22}\left(0\right)={p}_{23}\left(0\right)=0$ and $\:{p}_{24}\left(0\right)=1$ .

After obtaining the transient state probabilities of the Gearbox at different four states, the $\:{L}_{z}$*-*transform of the same element can be determined by employing the maximum tolerable shock in the Eq. (1) and subsequently substituting the corresponding values from Tables 2 and 3 as:

$$\begin{gathered} L_{z} \left( {G_{2} \left( t \right)} \right)\mathop \sum \limits_{{j = 1}}^{4} p_{{2j}} \left( t \right)z^{{\{ g_{{2j}} ,~{\delta }_{{2j}} \} ~~}} \hfill \\ = ~p_{{21}} \left( t \right)z^{{\left\{ {g_{{21}} ,~{\delta }_{{21}} } \right\}}} + p_{{22}} \left( t \right)z^{{\left\{ {g_{{22}} ,~{\delta }_{{22}} } \right\}}} + p_{{23}} \left( t \right)z^{{\left\{ {g_{{23}} ,~{\delta }_{{23}} } \right\}}} + ~p_{{24}} \left( t \right)z^{{\left\{ {g_{{24}} ,~{\delta }_{{24}} } \right\}}} \hfill \\ = ~p_{{21}} \left( t \right)z^{{\left\{ {0,~~0} \right\}}} + p_{{22}} \left( t \right)z^{{\left\{ {20,~~1.1} \right\}}} + p_{{23}} \left( t \right)z^{{\left\{ {34,~~1.9} \right\}}} + p_{{24}} \left( t \right)z^{{\left\{ {48,~~2.6} \right\}}} \hfill \\ \end{gathered}$$

**Fig. 7:**
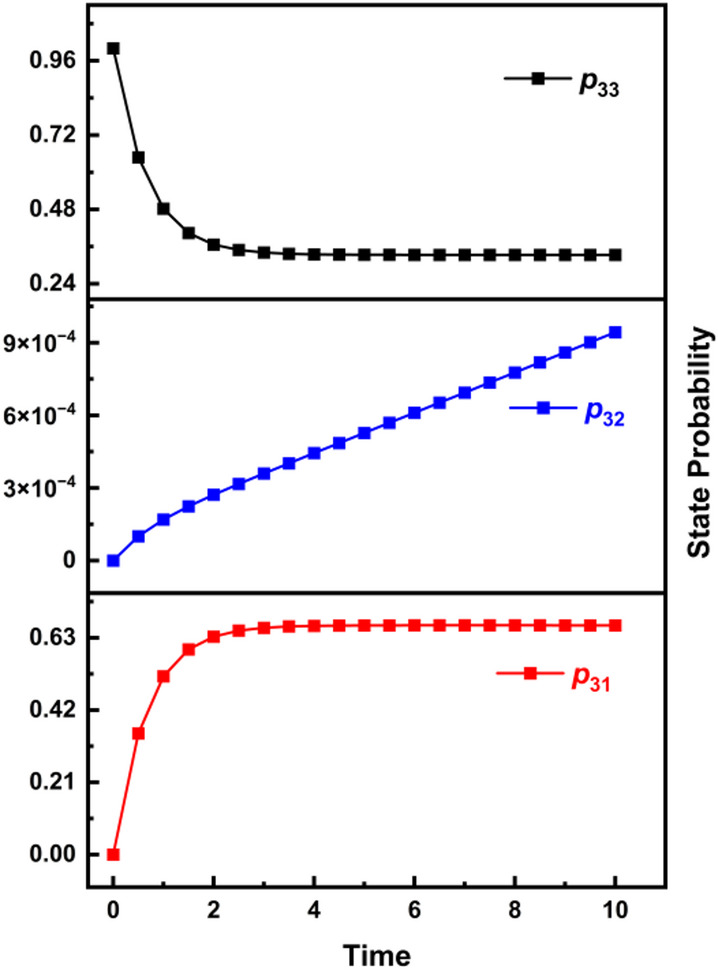
Transient probabilities of the generator.

**Fig. 8:**
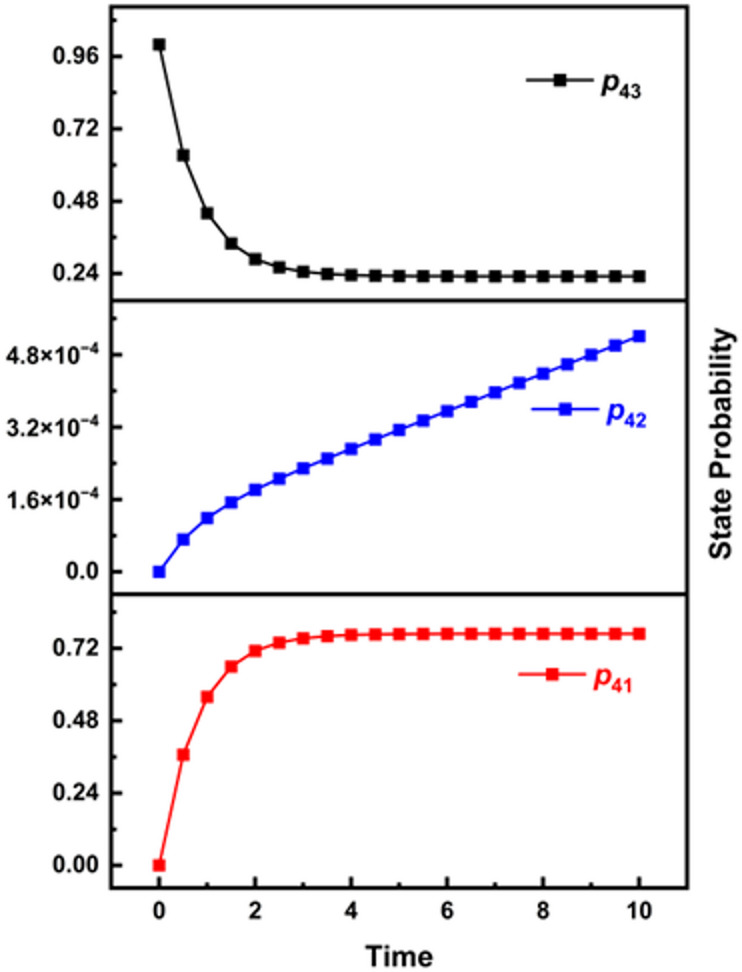
Transient probabilities of the converter.

**Fig. 9:**
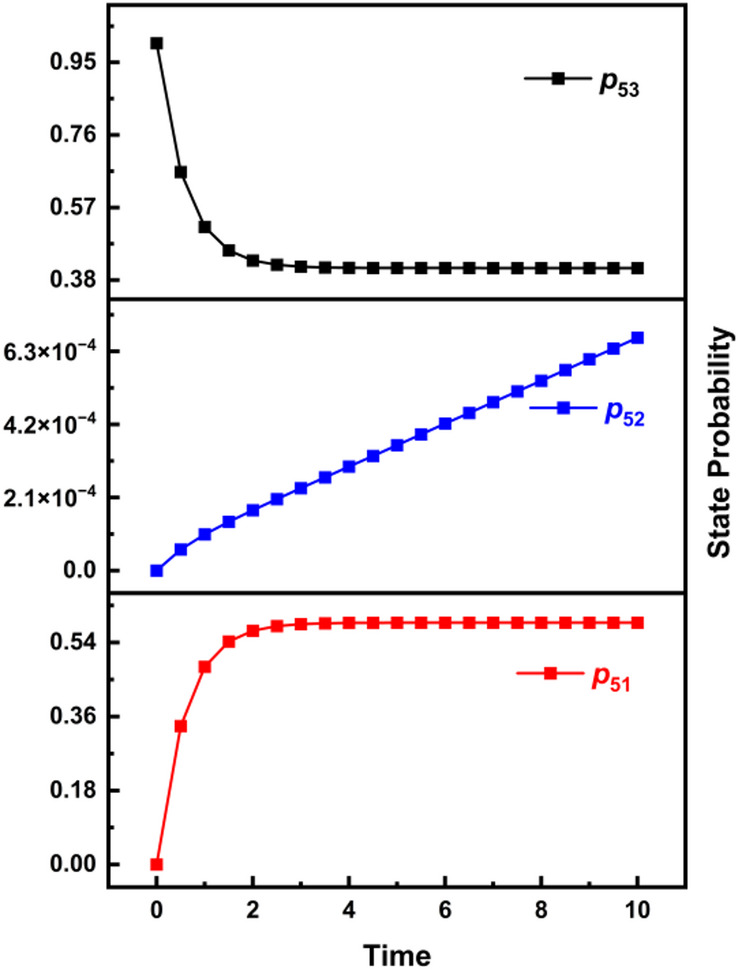
Transient probabilities of the transformer.

Following the same methodology is applied to the first two components, a set of differential equations can be developed to model the combined Markov degradation and external shock of the third, fourth, and fifth Doubly-Fed Induction Generator (DFIG) components: the Generator, Converter, and Transformer. Each of these three multi-state elements (MEs) is defined by three distinct states: State 3 (optimal performance), State 2 (intermediate degradation), and State 1 (complete failure). The system’s response to external shocks is state-dependent. If the intensity of a shock remains below the maximum tolerable threshold, the component continues its gradual Markov degradation. However, a shock exceeding this threshold while the component is in State 3 will cause an immediate transition to the failure state (State 1). This catastrophic transition is governed by the transition rate $\:{e}^{{\lambda\:}_{31}t}$. The resulting systems of differential equations are solved using the initial conditions $\:{p}_{i3}\left(0\right)=1$ and $\:{p}_{i1}\left(0\right)={p}_{i2}\left(0\right)=0\:$for $\:i=3,\:4,\:5$ (representing the Generator, Converter, and Transformer, respectively). The time-dependent state probability solutions are presented in Figs. 7 and 8, and 9.

After obtaining the state probabilities for the DFIG’s generator, converter, and transformer, the extended $\:{L}_{z}$-transform for the proposed shock model can be obtained as:

$$\begin{gathered} L_{z} \left( {G_{3} \left( t \right)} \right)\mathop \sum \limits_{{j = 1}}^{3} p_{{3j}} \left( t \right)z^{{\{ g_{{3j}} ,~{\delta }_{{3j}} \} ~~}} \hfill \\ = ~p_{{31}} \left( t \right)z^{{\left\{ {g_{{31}} ,~{\delta }_{{31}} } \right\}}} + p_{{32}} \left( t \right)z^{{\left\{ {g_{{32}} ,~{\delta }_{{32}} } \right\}}} + p_{{33}} \left( t \right)z^{{\left\{ {g_{{33}} ,~{\delta }_{{33}} } \right\}}} \hfill \\ ~~ = ~p_{{31}} \left( t \right)z^{{\left\{ {0,~~0} \right\}}} + p_{{32}} \left( t \right)z^{{\left\{ {20,~~2.0} \right\}}} + p_{{33}} \left( t \right)z^{{\left\{ {40,~~3.0} \right\}}} \hfill \\ \end{gathered}$$

$$\begin{gathered} L_{z} \left( {G_{4} \left( t \right)} \right)\mathop \sum \limits_{{j = 1}}^{3} p_{{4j}} \left( t \right)z^{{\{ g_{{4j}} ,~{\delta }_{{4j}} \} ~~}} \hfill \\ = ~p_{{41}} \left( t \right)z^{{\left\{ {g_{{41}} ,~{\delta }_{{41}} } \right\}}} + p_{{42}} \left( t \right)z^{{\left\{ {g_{{42}} ,~{\delta }_{{42}} } \right\}}} + p_{{43}} \left( t \right)z^{{\left\{ {g_{{43}} ,~{\delta }_{{43}} } \right\}}} \hfill \\ = ~p_{{41}} \left( t \right)z^{{\left\{ {0,~~0} \right\}}} + p_{{42}} \left( t \right)z^{{\left\{ {23,~~1.7} \right\}}} + p_{{43}} \left( t \right)z^{{\left\{ {52,~~2.8} \right\}}} \hfill \\ \end{gathered}$$

$$\begin{gathered} L_{z} \left( {G_{5} \left( t \right)} \right)~\mathop \sum \limits_{{j = 1}}^{3} p_{{5j}} \left( t \right)z^{{\{ g_{{5j}} ,~{\delta }_{{5j}} \} ~~}} \hfill \\ = ~p_{{51}} \left( t \right)z^{{\left\{ {g_{{51}} ,~\user2{\delta }_{{51}} } \right\}}} + p_{{52}} \left( t \right)z^{{\left\{ {g_{{52}} ,~{\delta }_{{52}} } \right\}}} + p_{{53}} \left( t \right)z^{{\left\{ {g_{{53}} ,~{\delta }_{{53}} } \right\}}} \hfill \\ ~ = ~p_{{51}} \left( t \right)z^{{\left\{ {0,~~0} \right\}}} + p_{{52}} \left( t \right)z^{{\left\{ {28,~~0.7} \right\}}} + p_{{53}} \left( t \right)z^{{\left\{ {55,~~2.5} \right\}}} \hfill \\ \end{gathered}$$

After obtaining the $\:{L}_{z}$-transform for each of the five DFIG components, the overall system’s transform for the combined shock and degradation model is computed by integrating these subsystems via Eq. (2). The reliability block diagram (Fig. 4) shows that the Generator and Converter are connected in series; their combined $\:{L}_{z}$-transform is therefore derived as follows:

Moreover, these two components are first combined in parallel. This parallel combination is then connected in series with the Blade, the Gearbox, and the Transformer. Mathematically, the $\:{L}_{z}$-transform of the entire DFIG system can be expressed as:

$$\begin{gathered} {{\Omega }}_{{f_{{series}} }} \left\{ {L_{z} \left( {G_{3} \left( t \right)} \right),~L_{z} \left( {G_{4} \left( t \right)} \right)} \right\} = {{\Omega }}_{{f_{{series}} }} \left\{ {\begin{array}{*{20}c} {\left( {p_{{31}} \left( t \right)z^{{\left\{ {0,~~0} \right\}}} + p_{{32}} \left( t \right)z^{{\left\{ {20,~~2.0} \right\}}} + p_{{33}} \left( t \right)z^{{\left\{ {40,~~3.0} \right\}}} } \right),} \\ {\left( {p_{{41}} \left( t \right)z^{{\left\{ {0,~~0} \right\}}} + p_{{42}} \left( t \right)z^{{\left\{ {23,~~1.7} \right\}}} + p_{{43}} \left( t \right)z^{{\left\{ {52,~~2.8} \right\}}} } \right)} \\ \end{array} } \right\} \hfill \\ = p_{{31}} \left( t \right)p_{{41}} \left( t \right)z^{{\left\{ {0,~~0} \right\}}} + p_{{31}} \left( t \right)p_{{42}} \left( t \right)z^{{\left\{ {0,~~1.7} \right\}}} + p_{{31}} \left( t \right)p_{{43}} \left( t \right)z^{{\left\{ {0,~~2.8} \right\}}} \hfill \\ + p_{{32}} \left( t \right)p_{{41}} \left( t \right)z^{{\left\{ {0,~~2.0} \right\}}} + p_{{32}} \left( t \right)p_{{42}} \left( t \right)z^{{\left\{ {20,~~3.7} \right\}}} + p_{{32}} \left( t \right)p_{{43}} \left( t \right)z^{{\left\{ {20,~~4.8} \right\}}} \hfill \\ + p_{{33}} \left( t \right)p_{{41}} \left( t \right)z^{{\left\{ {0,~~3.0} \right\}}} + p_{{33}} \left( t \right)p_{{42}} \left( t \right)z^{{\left\{ {23,~~4.7} \right\}}} + p_{{33}} \left( t \right)p_{{43}} \left( t \right)z^{{\left\{ {40,~~5.8} \right\}}} \hfill \\ \end{gathered}$$

6 $$\:L_{z} \left( {G_{{DFIG}} \left( t \right)} \right) = {\mathrm{O}}_{{f_{{series}} }} \left\{ {\begin{array}{*{20}c} {\left( {p_{{11}} \left( t \right)z^{{\left\{ {0,\:\:0} \right\}}} + p_{{12}} \left( t \right)z^{{\left\{ {15,\:\:0.8} \right\}}} + p_{{13}} \left( t \right)z^{{\left\{ {30,\:\:1.7} \right\}}} + p_{{14}} \left( t \right)z^{{\left\{ {50,\:\:2.2} \right\}}} } \right),} \\ {\:\left( {p_{{21}} \left( t \right)z^{{\left\{ {0,\:\:0} \right\}}} + p_{{22}} \left( t \right)z^{{\left\{ {20,\:\:1.1} \right\}}} + p_{{23}} \left( t \right)z^{{\left\{ {34,\:\:1.9} \right\}}} + p_{{24}} \left( t \right)z^{{\left\{ {48,\:\:2.6} \right\}}} } \right),} \\ {\:{\mathrm{O}}_{{f_{{Parallel}} }} \left( {\begin{array}{*{20}c} {\left( {\begin{array}{*{20}c} {p_{{31}} \left( t \right)p_{{41}} \left( t \right)z^{{\left\{ {0,\:\:0} \right\}}} + p_{{31}} \left( t \right)p_{{42}} \left( t \right)z^{{\left\{ {0,\:\:1.7} \right\}}} + p_{{31}} \left( t \right)p_{{43}} \left( t \right)z^{{\left\{ {0,\:\:2.8} \right\}}} } \\ {\: + p_{{32}} \left( t \right)p_{{41}} \left( t \right)z^{{\left\{ {0,\:\:2.0} \right\}}} + p_{{32}} \left( t \right)p_{{42}} \left( t \right)z^{{\left\{ {20,\:\:3.7} \right\}}} + p_{{32}} \left( t \right)p_{{43}} \left( t \right)z^{{\left\{ {20,\:\:4.8} \right\}}} } \\ {\: + p_{{33}} \left( t \right)p_{{41}} \left( t \right)z^{{\left\{ {0,\:\:3.0} \right\}}} + p_{{33}} \left( t \right)p_{{42}} \left( t \right)z^{{\left\{ {23,\:\:4.7} \right\}}} + p_{{33}} \left( t \right)p_{{43}} \left( t \right)z^{{\left\{ {40,\:\:5.8} \right\}}} } \\ \end{array} } \right),} \\ {\:\left( {\begin{array}{*{20}c} {p_{{31}} \left( t \right)p_{{41}} \left( t \right)z^{{\left\{ {0,\:\:0} \right\}}} + p_{{31}} \left( t \right)p_{{42}} \left( t \right)z^{{\left\{ {0,\:\:1.7} \right\}}} + p_{{31}} \left( t \right)p_{{43}} \left( t \right)z^{{\left\{ {0,\:\:2.8} \right\}}} } \\ {\: + p_{{32}} \left( t \right)p_{{41}} \left( t \right)z^{{\left\{ {0,\:\:2.0} \right\}}} + p_{{32}} \left( t \right)p_{{42}} \left( t \right)z^{{\left\{ {20,\:\:3.7} \right\}}} + p_{{32}} \left( t \right)p_{{43}} \left( t \right)z^{{\left\{ {20,\:\:4.8} \right\}}} } \\ {\: + p_{{33}} \left( t \right)p_{{41}} \left( t \right)z^{{\left\{ {0,\:\:3.0} \right\}}} + p_{{33}} \left( t \right)p_{{42}} \left( t \right)z^{{\left\{ {23,\:\:4.7} \right\}}} + p_{{33}} \left( t \right)p_{{43}} \left( t \right)z^{{\left\{ {40,\:\:5.8} \right\}}} } \\ \end{array} } \right)} \\ \end{array} } \right)\prime \:} \\ {\:\left( {p_{{51}} \left( t \right)z^{{\{ 0,\:\:0\} }} + p_{{52}} \left( t \right)z^{{\{ 28,\:\:0.7\} }} + p_{{53}} \left( t \right)z^{{\{ 55,\:\:2.5\} }} } \right)} \\ \end{array} } \right\}$$

After the $\:{L}_{z}$-transform of the entire DFIG is obtained by expanding the Eq. (6), its performance measures can be obtained from the same. These performance measures include time-dependent reliability, availability and mean expected performance, which serves as the backbone of performance associated with any system.

#### Dynamic availability of DFIG

The time-dependent availability, $\:A\left(t\right)$, of a Doubly-Fed Induction Generator (DFIG) is defined as the probability that the system is operational at a given time * t*, considering its failure and repair processes. It is derived from the system’s reliability model through the $\:{L}_{z}$-transform $\:\left({L}_{z}\left({G}_{DFIG}\left(t\right)\right)\:\right)$ and reflects the instantaneous capability of the DFIG to perform its intended function. Mathematically, the time-dependent availability ($\:A\left(t\right))$ for the proposed model of the DFIG for the minimum required performance * K* can be expressed as:

7 $$A\left( t \right) = ~\mathop \sum \limits_{j} p_{{ij}} \left( t \right){\mathrm{~}}.\beta \left( {\Phi _{{{\mathrm{DFIG}}}} \left( {g_{{ij_{1} }} ,g_{{ij_{{2, \ldots ... .,}} }} g_{{ij_{{n - 1}} }} ,g_{{ij_{n} }} } \right) - K} \right)$$

where, $\:{p}_{ij}\left(t\right)$ is the transient state probability of the $\:{i}^{th}$ component of DFIG at state * j* and the function $\:\beta\:\left(y\right)$ is given by $$\:\beta\:\left(y\right)=\left\{\begin{array}{c}1,\:\:\:y\ge\:0\\\:0,\:\:y<0\end{array}\right.$$.

**Fig. 10:**
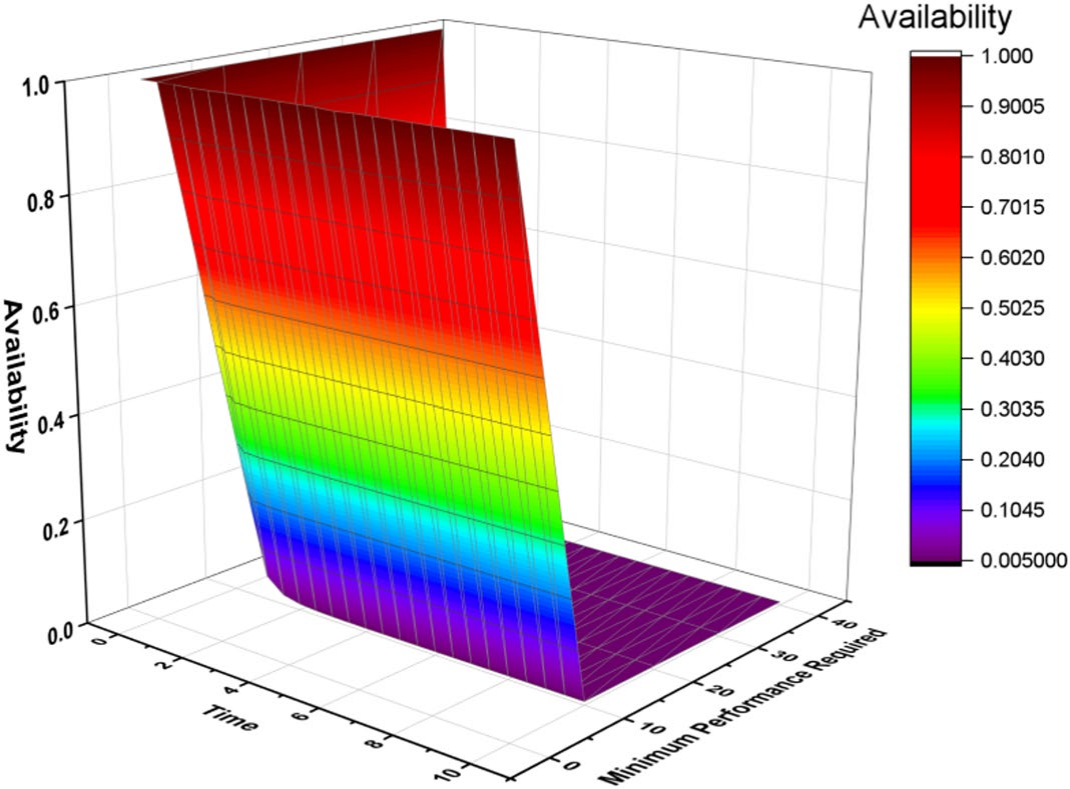
Time-dependent availability of DFIG for combined external extreme shock and internal degradation.

The time-dependent availability of the DFIG, accounting for combined shock and degradation processes, is computed using Eqs. (6) and (7) in conjunction with the state probabilities illustrated in Figs. 5, 6, 7, 8 and 9. The analysis spans a time range from $\:t\:=\:$0 to 10 units and a system performance range from 0 to 40 units. The resulting availability values are graphically represented in Fig. 10.

#### Dynamic reliability of DFIG

The time-dependent reliability, $\:R\left(t\right)$, of DFIG represents the probability that the system operates without failure up to time * t*, under combined shock and degradation processes. In this case, the state probabilities are obtained considering its failure process, not its repair process. Mathematically, the time-dependent reliability ($\:R\left(t\right))$ for the proposed model of the DFIG for the minimum required performance * K* can be expressed as:

8 $$\:R\left(t\right)=\:\sum\:_{j}{p}_{ij}\left(t\right)\:.\alpha\:\left({\varPhi\:}_{\mathrm{D}\mathrm{F}\mathrm{I}\mathrm{G}}\left({g}_{{ij}_{1}},{g}_{{ij}_{2,\dots\:\dots\:\dots\:\dots\:.,}}{g}_{{ij}_{n-1}},{g}_{{ij}_{n}}\right)-K\right)$$

where, $\:{p}_{ij}\left(t\right)$ is the transient state probability of the $\:{i}^{th}$ component of DFIG at state * j* obtained by solving the system of differential equation for zero repair rate and failure rate specified in Table 2 and the function$\:\:\alpha\:\left(x\right)$ is given by $$\:\alpha\:\left(x\right)=\left\{\begin{array}{c}1,\:\:\:x\ge\:0\\\:0,\:\:x<0\end{array}\right.$$.

**Fig. 11:**
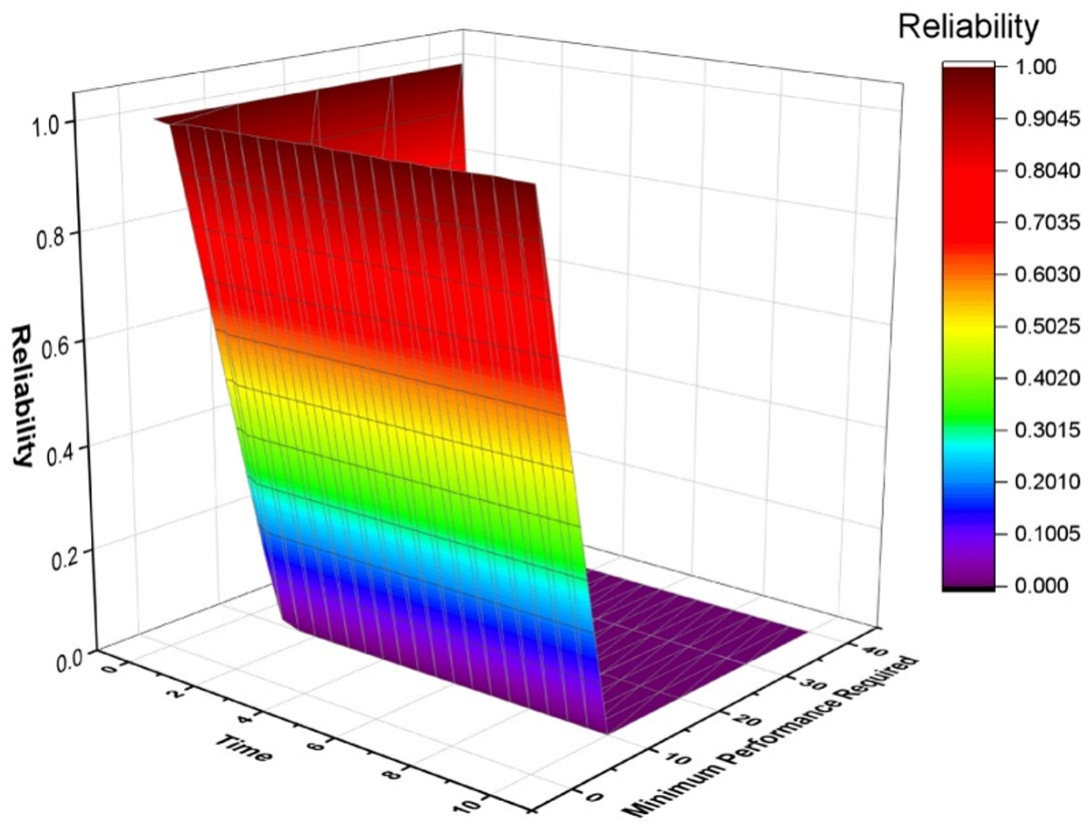
Time-dependent reliability of dfig for combined external extreme shock and internal degradation.

The time-dependent reliability $\:R\left(t\right)$ of the DFIG is derived using Eqs. (6) and (8), incorporating the state probabilities associated with system reliability. The analysis covers a temporal domain from $\:t=\:$0 to 10 units and a performance (* K*) range from 0 to 40 units. The resultant reliability values are visually depicted in Fig. 11.

### Performance measures of DFIG under proposed maintenance strategy

The system model comprises several critical components with distinct operational states. The Blade and Gearbox components of the DFIG are each modeled with four discrete states, while the Generator, Converter, and Transformer are each modeled with three states. For all components, the fully operational state (denoted as state 4 for the four-state models and state 3 for the three-state models) represents the best-performing condition. This section applies a novel maintenance strategy, as formally proposed in Sect.  4, to evaluate its impact on system reliability. The primary objective is to compare key time-dependent reliability indices against a baseline scenario where no maintenance is performed. The analysis incorporates the effect of external extreme shocks, which can occur randomly and impact the system irrespective of its current degradation state. Each state of every component possesses a specific shock tolerance threshold, defined as its maximum tolerable shock limit. A fundamental assumption is that a component in a higher, healthier state exhibits a greater ability to withstand external shocks compared to a lower, more degraded state. Furthermore, it is assumed that external shocks only strike a component when it is operating in one of its two best-performing states. Under the proposed maintenance strategy, if an external shock impacts the DFIG’s Blade component while it is in state 4, its response is modified. Upon the occurrence of an external shock, the component transitions to a dedicated dummy state, labeled 4′, irrespective of whether the shock magnitude exceeds the state’s tolerable limit. From this maintenance-affected state (4’), the component continues its internal Markovian degradation process. However, the transition intensity to the major failure state (state 1) is reduced from the original time-dependent rate, $\:{e}^{{\lambda\:}_{41}t}$, to a constant rate, $\:{\lambda\:}_{{4}^{{\prime\:}}1}$. Similar maintenance holds for State 3 and all other repair mechanisms and transition paths remain unchanged. The system of differential equations required to solve for the transient state probabilities of the Blade component, incorporating this maintenance strategy, is formulated as follows:

9 $$\begin{gathered} \frac{{dp_{{11}} \left( t \right)}}{{dt}} = - 0.6.p_{{11}} \left( t \right) + 0.00001.p_{{12}} \left( t \right) + 0.0000028.p_{{13^{{\mathrm{'}}} }} \left( t \right) + 0.000005.p_{{14^{{\mathrm{'}}} }} \left( t \right) \hfill \\ \frac{{dp_{{12}} \left( t \right)}}{{dt}} = - 0.00001.p_{{12}} \left( t \right) + 0.00004.p_{{13^{{\mathrm{'}}} }} \left( t \right) \hfill \\ \frac{{dp_{{13^{{\mathrm{'}}} }} \left( t \right)}}{{dt}} = - 0.0000428.p_{{13^{{\mathrm{'}}} }} \left( t \right) + 0.00007p_{{14^{{\mathrm{'}}} }} \left( t \right) \hfill \\ \frac{{dp_{{14^{{\mathrm{'}}} }} \left( t \right)}}{{dt}} = 0.6.p_{{11}} \left( t \right) - 0.000075.p_{{14^{{\mathrm{'}}} }} \left( t \right) \hfill \\ \end{gathered}$$

**Fig. 12:**
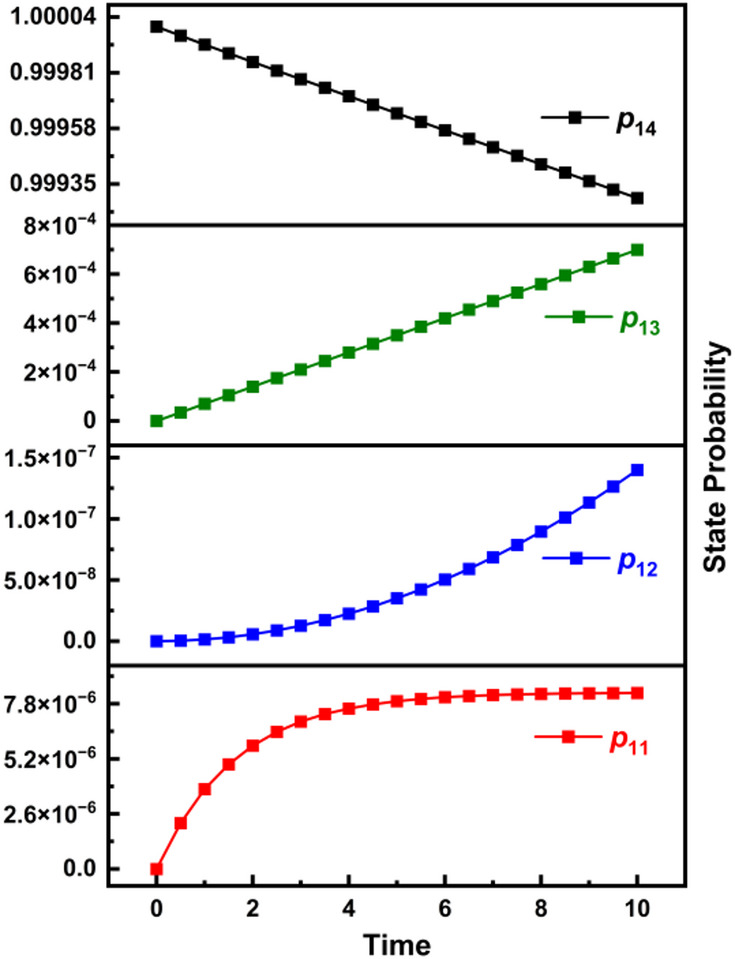
State probabilities of the blade under maintenance.

Now these system of differential equations are solved under initial conditions $\:{p}_{11}\left(0\right)={p}_{12}\left(0\right)={p}_{{13}^{{\prime\:}}}\left(0\right)=0$ and $\:{p}_{{14}^{{\prime\:}}}\left(0\right)=1$ to obtain the transient state probabilities as depicted in Fig. 12.

The $\:{L}_{z}$-transform of the first component (Blade) under maintenance with the help of dummy state is obtained by

$$L_{z} \left( {G_{1} \left( t \right)} \right)\: = \:p_{{11}} \left( t \right)z^{{\{ 0,\:\:0\} }} + p_{{12}} \left( t \right)z^{{\{ 15,\:\:0.8\} }} + p_{{13^{{\prime {\kern 1pt} }} }} \left( t \right)z^{{\{ 30,\:\:1.7\} }} + p_{{14^{{\prime {\kern 1pt} }} }} \left( t \right)z^{{\{ 50,\:\:2.2\} }}$$.

Similarly, the system of differential equations can be formed for remaining four components of DFIG as per maintenance strategy and solved under similar initial conditions to yield the transient state probabilities and is plotted w.r.t. as:

**Fig. 13:**
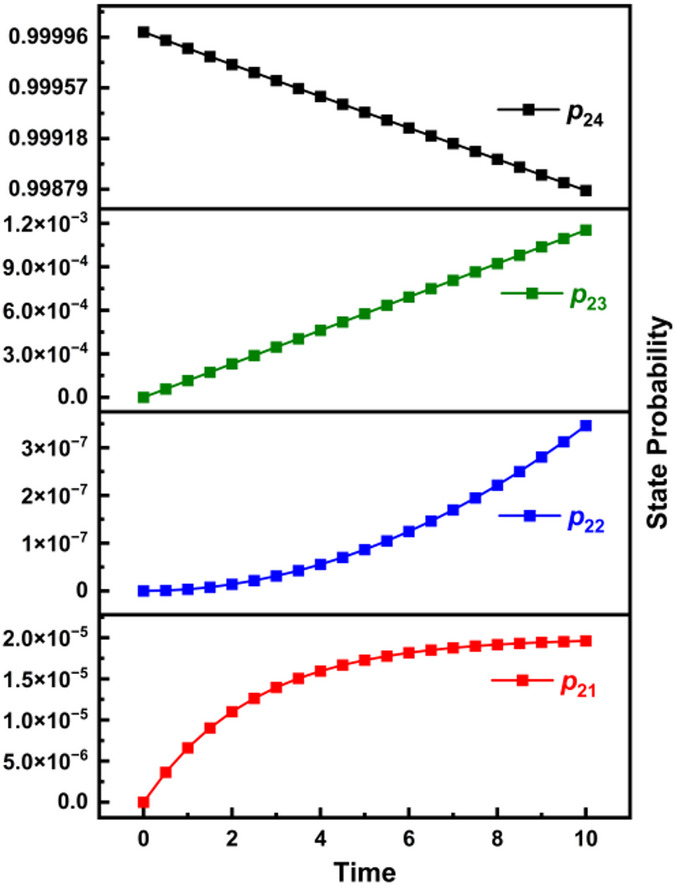
State probabilities of the gearbox under maintenancefigure.

**Fig. 14:**
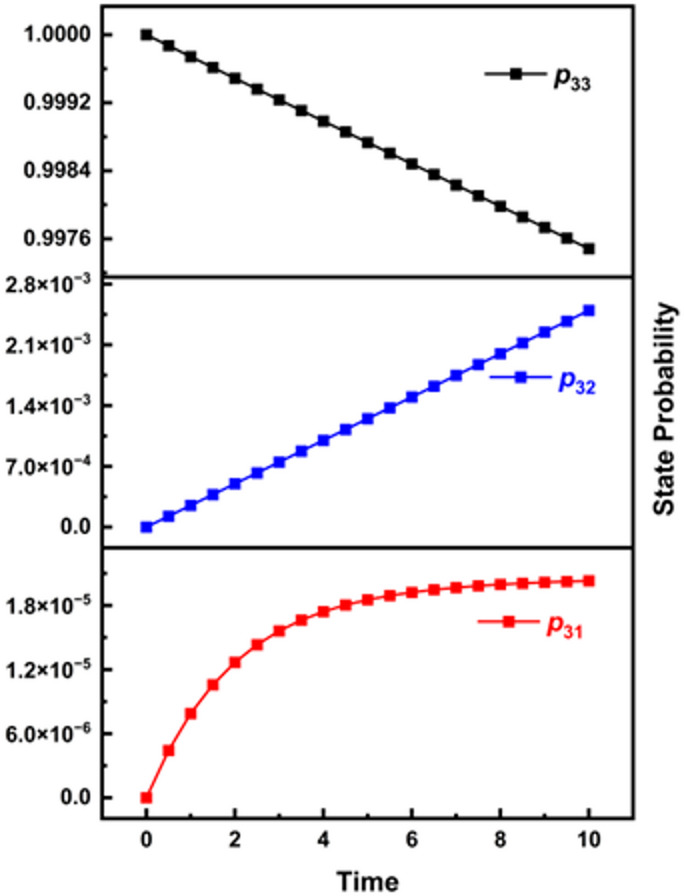
State probabilities of the generator under maintenance.

**Fig. 15:**
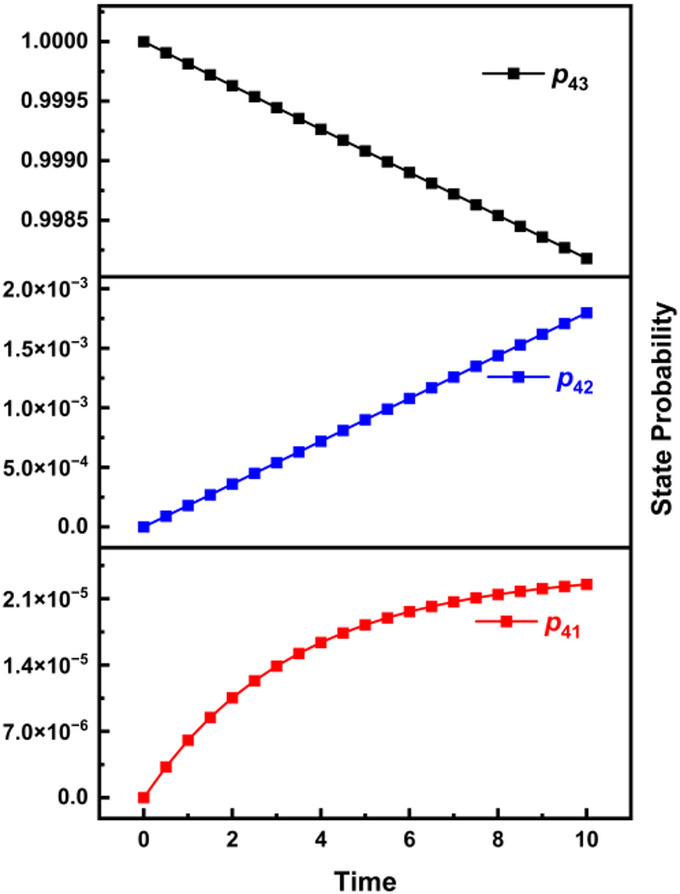
State probabilities of the convertor under maintenance.

**Fig. 16:**
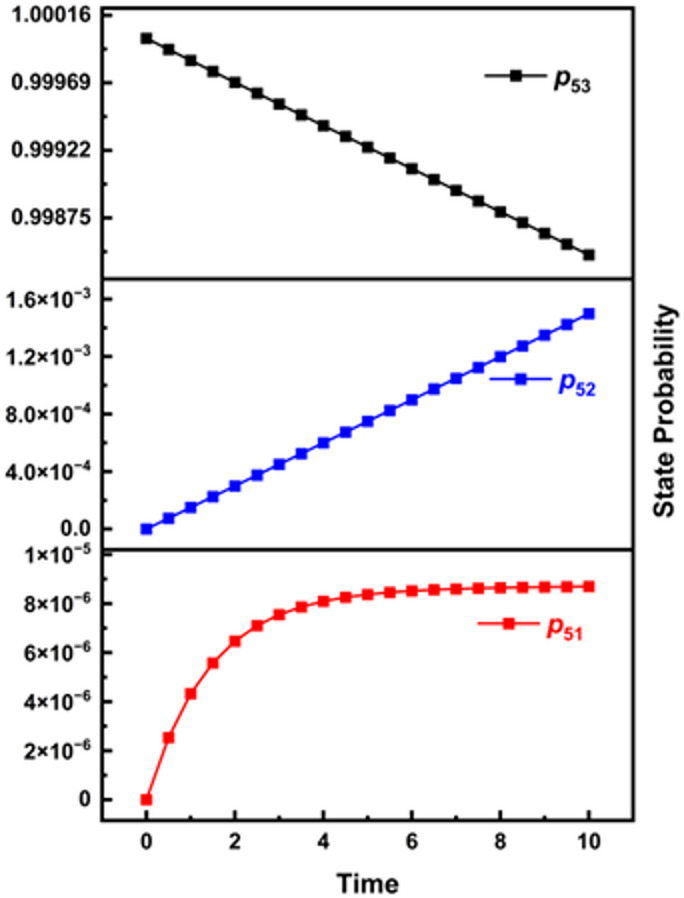
State probabilities of the transformer under maintenance.

Following the derivation of the state probabilities for each component under the specified maintenance strategy, the corresponding $\:{L}_{z}$-transforms are obtained.

$$L_{z} \left( {G_{2} \left( t \right)} \right)~ = ~p_{{21}} \left( t \right)z^{{\left\{ {0,~~0} \right\}}} + p_{{22}} \left( t \right)z^{{\left\{ {20,~~1.1} \right\}}} + p_{{23^{'} }} \left( t \right)z^{{\left\{ {34,~~1.9} \right\}}} + p_{{24^{'} }} \left( t \right)z^{{\left\{ {48,~~2.6} \right\}}}$$

$$\:L_{z} \left( {G_{3} \left( t \right)} \right) = ~p_{{31}} \left( t \right)z^{{\left\{ {0,~~0} \right\}}} + p_{{32^{'} }} \left( t \right)z^{{\left\{ {20,~~2.0} \right\}}} + p_{{33^{'} }} \left( t \right)z^{{\left\{ {40,~~3.0} \right\}}}$$

$$L_{z} \left( {G_{4} \left( t \right)} \right) = ~p_{{41}} \left( t \right)z^{{\left\{ {0,~~0} \right\}}} + p_{{42^{'} }} \left( t \right)z^{{\left\{ {23,~~1.7} \right\}}} + p_{{43^{'} }} \left( t \right)z^{{\left\{ {52,~~2.8} \right\}}}$$

$$\:{L}_{z}\left({G}_{5}\left(t\right)\right)\:=\:{p}_{51}\left(t\right){z}^{\{0,\:\:0\}}+{p}_{{52}^{{\prime\:}}}{\left(t\right)z}^{\{28,\:\:0.7\}}+{p}_{{53}^{{\prime\:}}}{\left(t\right)z}^{\{55,\:\:2.5\}}$$

These individual transforms are then combined using the composition operator in the similar way that of Eq. (6), which models the specific series-parallel structure of the DFIG system. This aggregation yields the $\:{L}_{z}$-transform for the entire system, for the state probabilities are illustrated in Figs. 12, 13, 14, 15 and 16. From this combined $\:{L}_{z}$-transform, the following dynamic reliability measures are derived, capturing the system’s behavior under the combined influence of internal degradation and external shocks.

#### Dynamic availability of DFIG under maintenance

**Fig. 17:**
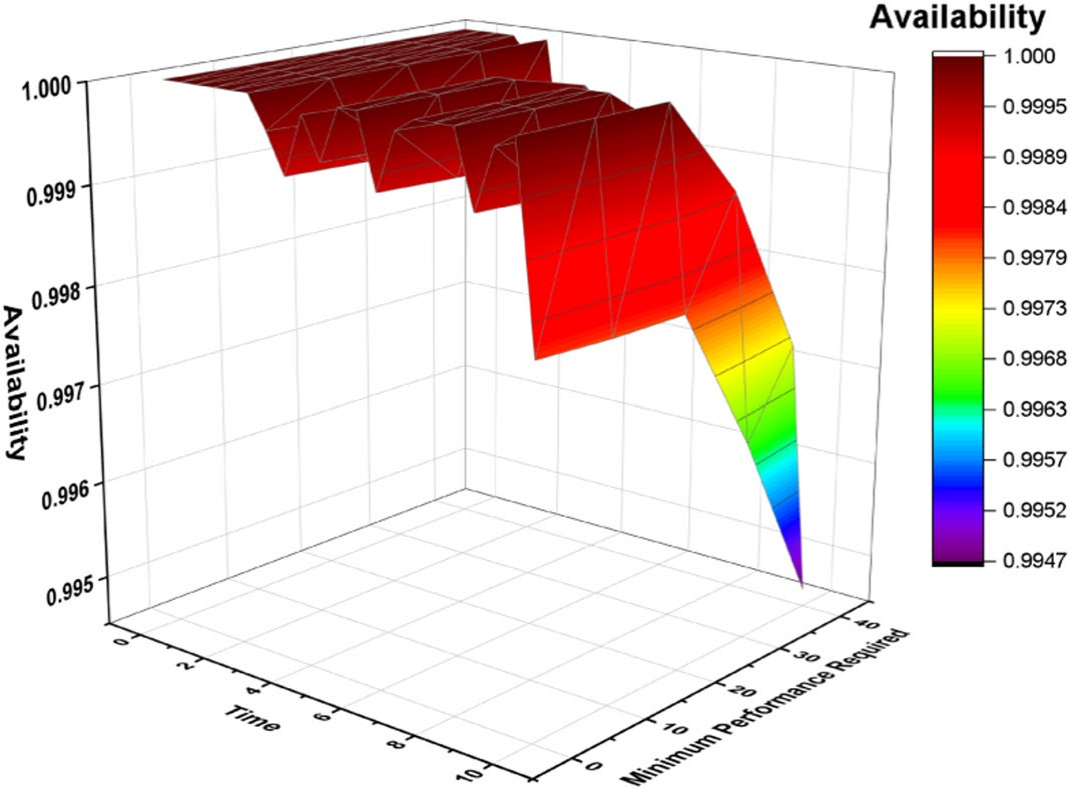
Time-dependent availability of DFIG under maintenance.

The dynamic or time-dependent availability of the DFIG under this maintenance strategy is derived by applying Eq. (7) to the system’s entire $\:{L}_{z}$-transform. Availability was computed over a time horizon of 10 units, with system performance levels ranging from 0 to 40 units. The results, plotted in Fig. 17, demonstrate a direct comparison of system availability with and without the maintenance strategy, clearly illustrating its significant positive impact on overall system availability.

The Fig. 17 demonstrate that system availability without maintenance declines significantly over time, especially as the minimum performance requirement increases. In contrast, implementing the proposed maintenance strategy ensures near-perfect availability (approximately 1.0) for the vast majority of the observed conditions. Maintenance effectively mitigates performance degradation, sustaining high system reliability even at higher performance thresholds and over extended durations.

#### Time-dependent reliability of DFIG under maintenance

**Fig. 18:**
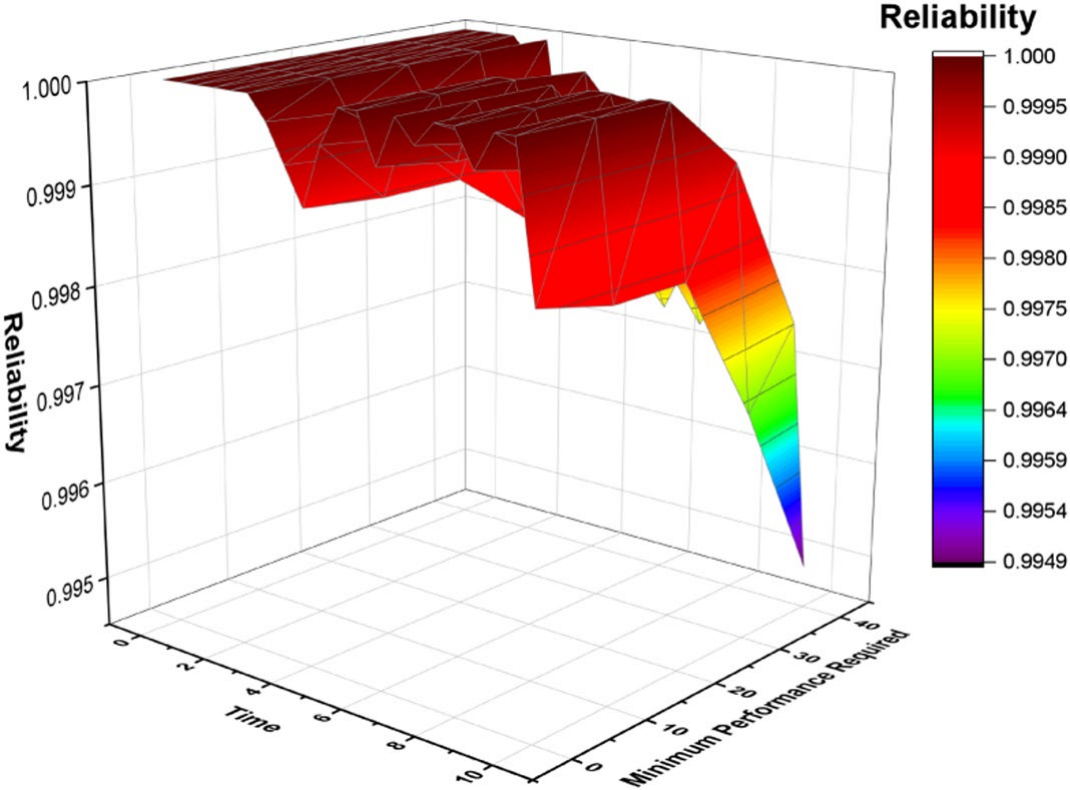
Time-dependent reliability of DFIG under maintenance.

In a corresponding manner, the system of differential equations governing reliability for this maintenance strategy is derived and solved under specified initial conditions to determine the transient state probabilities. Subsequently, the time-dependent reliability of the DFIG is computed utilizing Eq. (8) in conjunction with the complete $\:{L}_{z}$-transform of DFIG. For the reliability analysis, time is varied from 0 to 10 and system performance from 0 to 40; the results, which clearly demonstrate the positive impact of maintenance on the system’s overall reliability, are graphically presented in Fig. 18.

It shows that without maintenance, the system’s reliability drops extremely low very quickly, especially if a high-performance level is needed. However, with the maintenance strategy in place, the system’s reliability remains consistently perfect or near-perfect over the entire time and across all performance requirements. This proves that the maintenance plan is highly effective at preventing failure and keeping the system running reliably.

### Effect of varying shock tolerance on DFIG’s availability

This section examines the influence of varying shock tolerance thresholds on system availability within a combined shock-degradation model, under specified performance requirements. The analysis comparatively evaluates system behavior both in the presence and absence of a maintenance strategy. The primary objective is to quantitatively assess how different levels of robustness to external shocks impact overall availability, thereby providing a foundational understanding for the development of optimized design and maintenance protocols aimed at enhancing system resilience and sustaining operational performance.

**Fig. 19:**
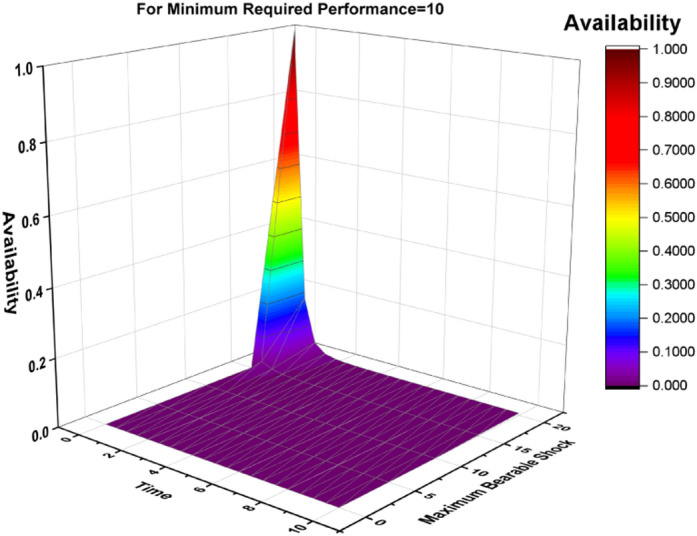
Availability of DFIG w.r.t. varying maximum tolerable shock for $\:\boldsymbol{K}=10$.

**Fig. 20:**
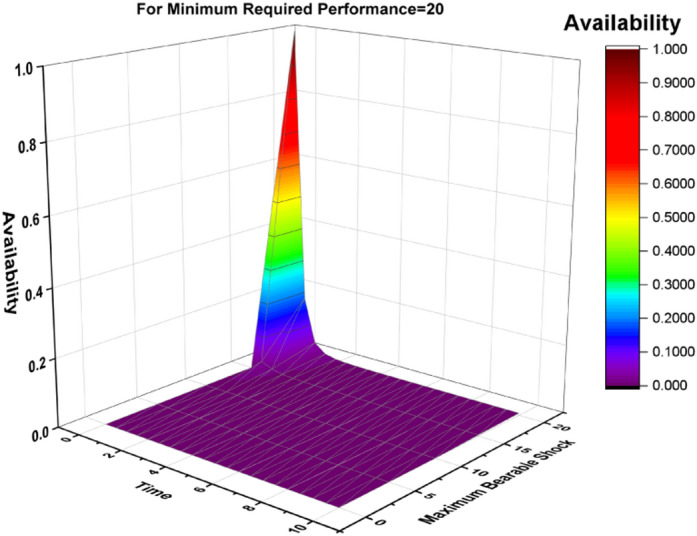
Availability of DFIG w.r.t. varying maximum tolerable shock for $\:\boldsymbol{K}=20$.

**Fig. 21:**
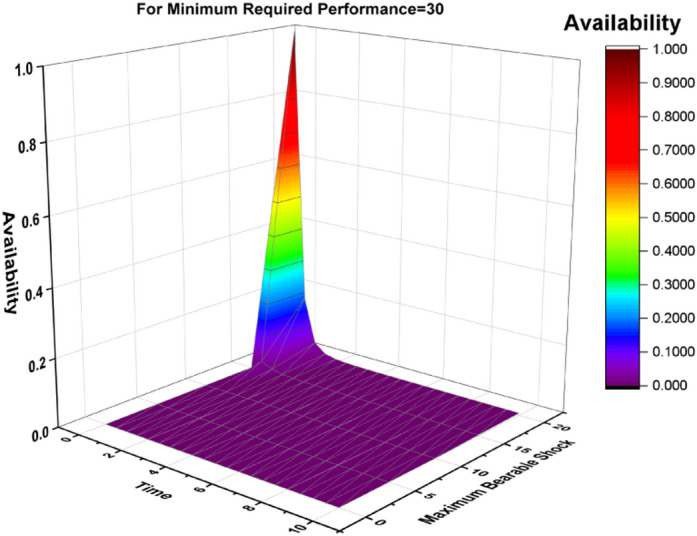
Availability of DFIG w.r.t. varying maximum tolerable shock for $\:\boldsymbol{K}=30$.

**Fig. 22:**
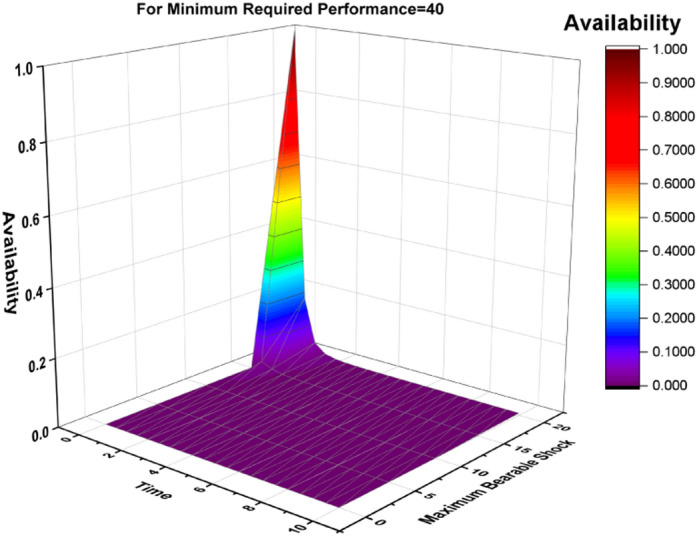
Availability of DFIG w.r.t. varying maximum tolerable shock for $\:\boldsymbol{K}=40$.

The availability of the DFIG (without maintenance) under varying shock tolerance thresholds, for a specified system performance level, is calculated using Eq. (6), by substituting the transient state probabilities of its components illustrated in Figs. 5, 6, 7, 8 and 9. For this analysis, the system performance level is held constant while the maximum tolerable shock threshold is assumed to be varied from 0 to 20 units, with time simultaneously varied from 0 to 10 units as depicted in Figs. 19, 20, 21 and 22.

The Figs. 19, 20, 21 and 22 represent the system availability over time under varying shock thresholds and redundancy levels$\:\:\left(K\right)$. The analysis reveals that the maximum bearable shock is the primary determinant of system survival, with availability being perfect (1.00) only at the highest shock threshold of 20. The effect of increasing redundancy (*K* from 10 to 40) is negligible, as availability values are identical across all *K* levels for any given time and maximum tolerable shock, indicating redundant components offer no protection against the shock-driven failures modeled. Furthermore, systems with a lower shock threshold (shock threshold = 15) exhibit a rapid decrease in availability early in the timeline before stabilizing at a low, constant probability of failure, suggesting an initial phase of high vulnerability.

#### Impact of shock tolerance threshold on DFIG availability under maintenance

**Fig. 23:**
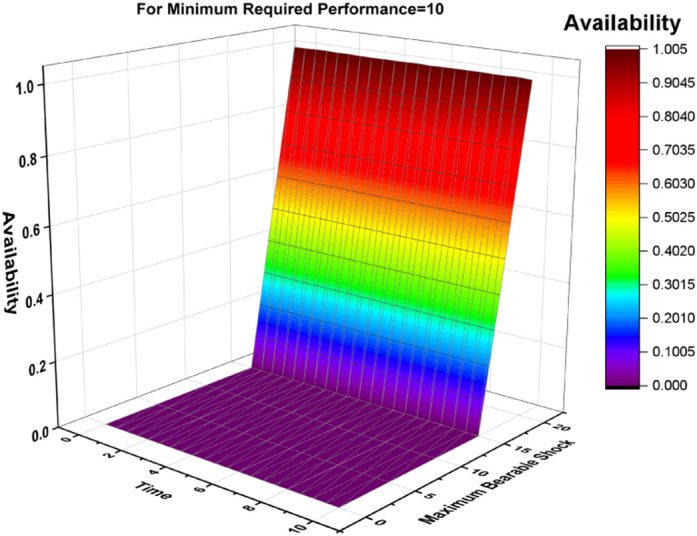
Availability of DFIG under maintenance w.r.t. varying maximum tolerable shock for $\:\boldsymbol{K}=10$.

**Fig. 24:**
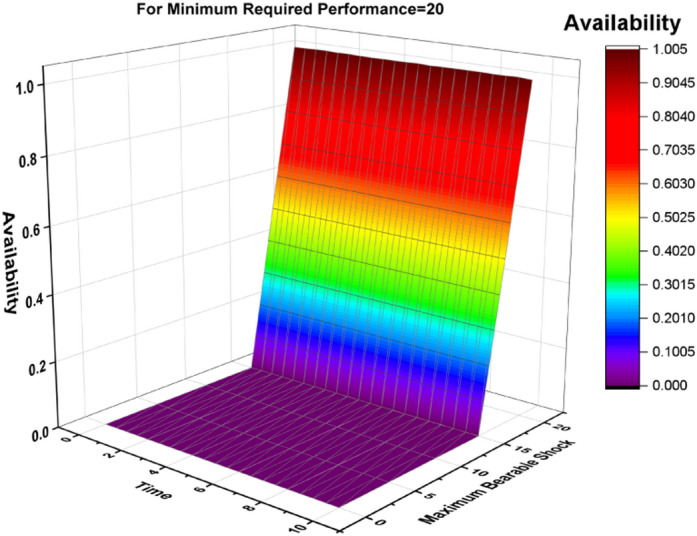
Availability of DFIG under maintenance w.r.t. varying maximum tolerable shock for $\:\boldsymbol{K}=20$.

**Fig. 25:**
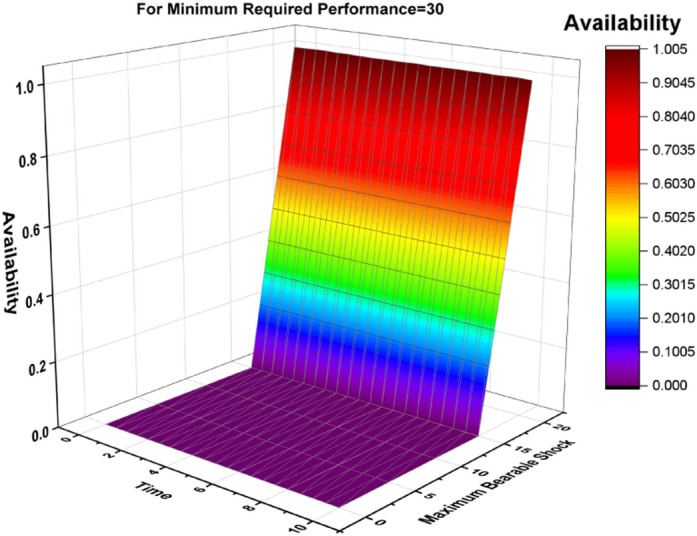
Availability of DFIG under maintenance w.r.t. varying maximum tolerable shock for $\:\boldsymbol{K}=30$.

**Fig. 26:**
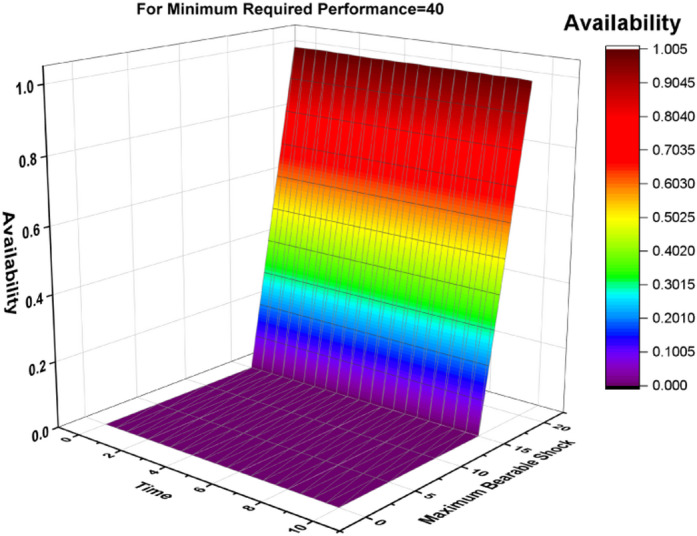
Availability of DFIG under maintenance w.r.t. varying maximum tolerable shock for $\:\boldsymbol{K}=40$.

This section analyzes the influence of the maximum shock tolerance threshold on the time-varying availability of a DFIG operating under a prescribed maintenance strategy. The core aim is to evaluate the efficacy of maintenance in preserving system availability across a spectrum of shock tolerance limits. The DFIG’s availability is computed via Eq. (6), which incorporates the transient state probabilities of its components (Figs. 12, 13, 14, 15 and 16), for a fixed performance level. The analysis involves varying the maximum shock threshold from 0 to 20 units and time from 0 to 10 units, with the results visualized in Figs. 23, 24, 25 and 26.

### Mean expected performance

10 $$E\left( t \right) = \mathop \sum \limits_{{\begin{array}{*{20}c} {j = 1} \\ {g_{{ij}} > 0} \\ \end{array} }}^{M} p_{{ij}} \left( t \right)g_{{ij}}$$

**Fig. 27:**
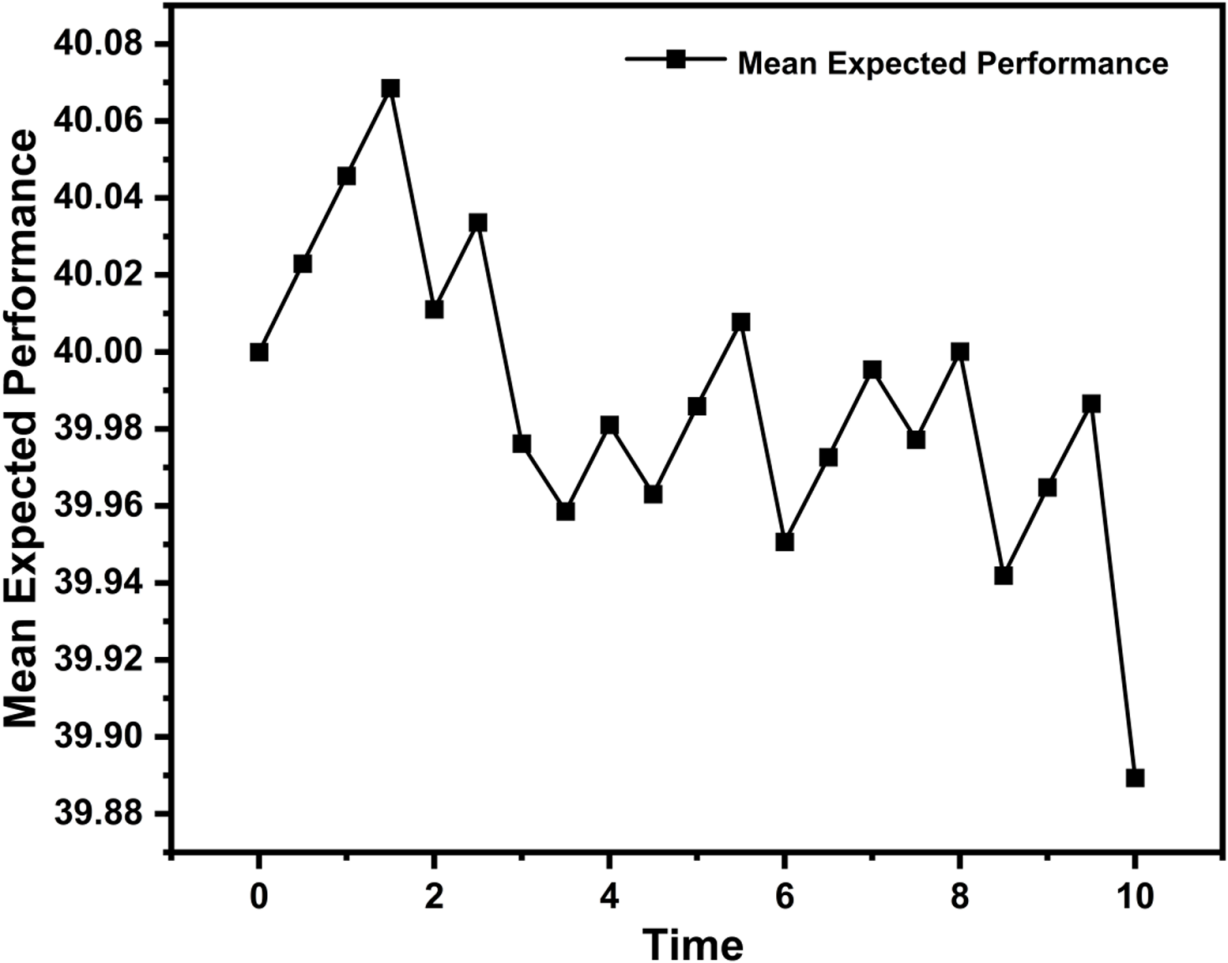
Mean expected performance.

For the state probabilities and performance level given in Table 2, the Mean Expected Performance can be obtained and is plotted in Fig. 27 as:

### Cost analysis

Assuming the service facility remains continuously accessible, the anticipated profit over the time span [0, t) is calculated as:

11 $$\:E_{p} \left( t \right) = c_{r} \mathop {\mathop {\int \: }\limits^{t} }\limits_{0} A\left( t \right) - c_{s} t$$

**Fig. 28 A:**
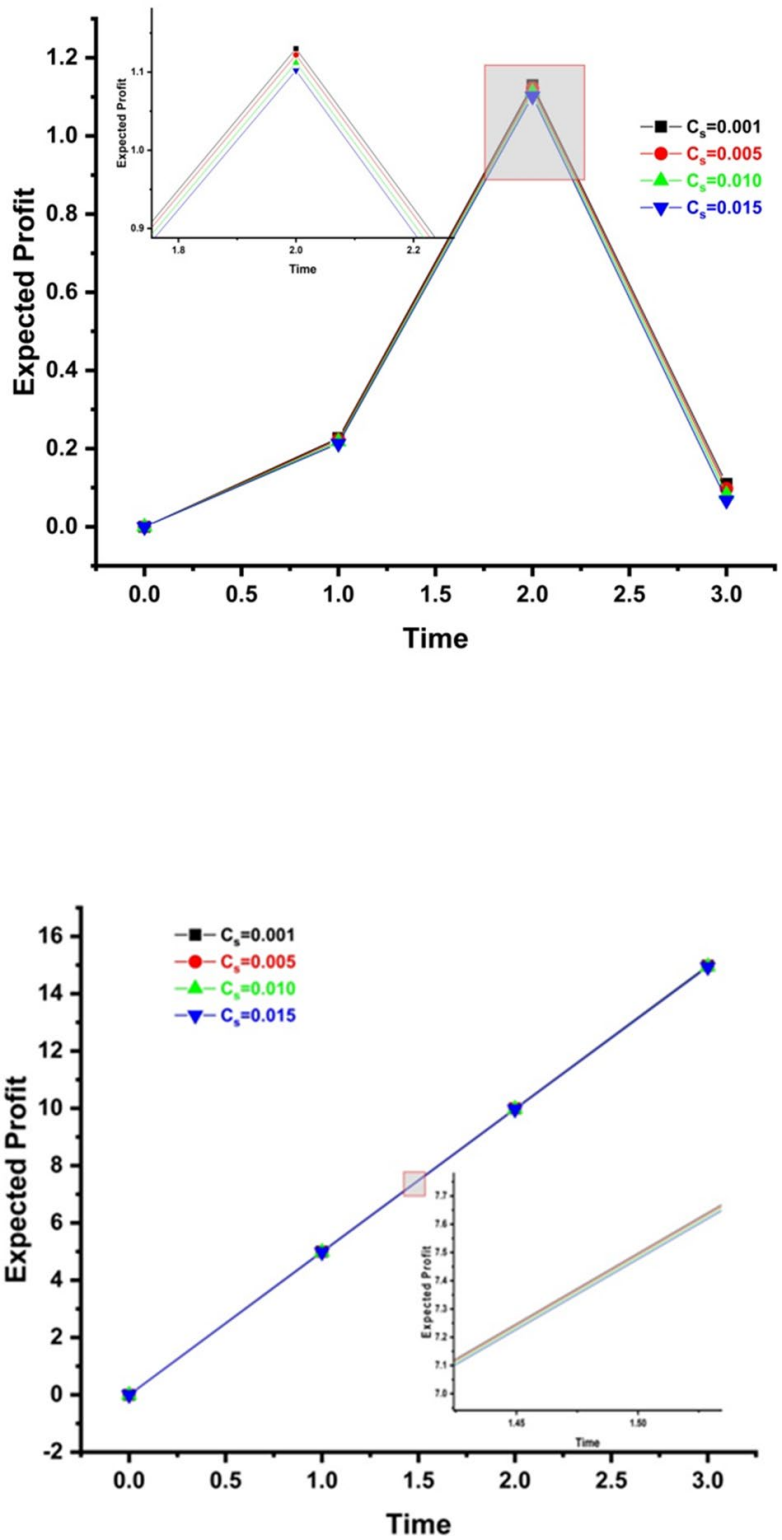
Expected Profit for $\:\boldsymbol{K}\:=\:40$ before maintenance **B**: Expected Profit for $\:\boldsymbol{K}\:=\:40$ After Maintenance.

Here, $\:{c}_{r}$ represents the revenue cost and $\:{c}_{s}\:$denotes the service cost. For the DFIG system, the repair cost was fixed at 5, while the service cost was varied across values of 0.001, 0.005, 0.01, and 0.015 to estimate the expected profit for $\:K\:=\:40$, with the results plotted in Fig. 28A and B.

**Fig. 29:**
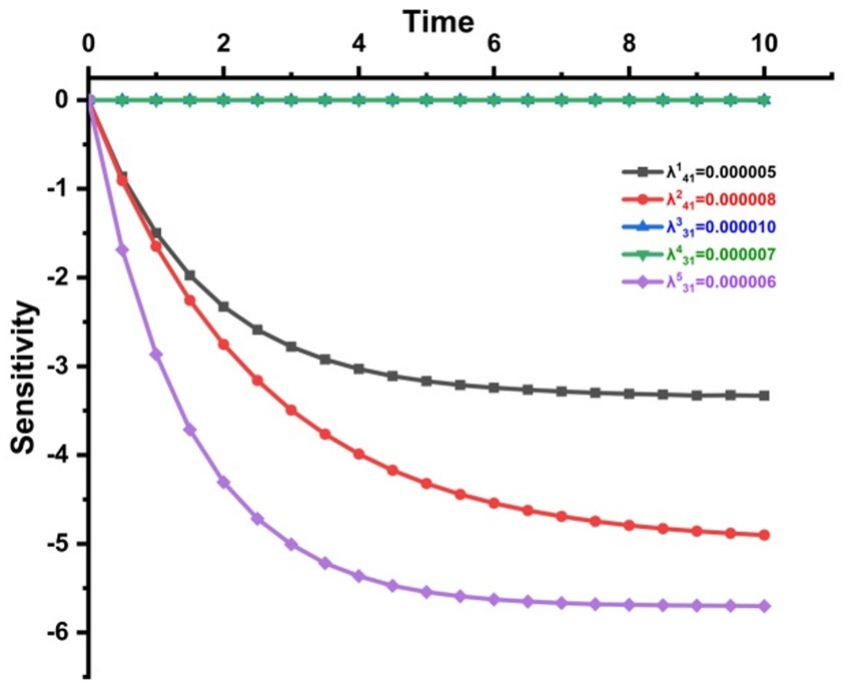
Sensitivity of DFIG for $\:\boldsymbol{K}=40$.

**Fig. 30:**
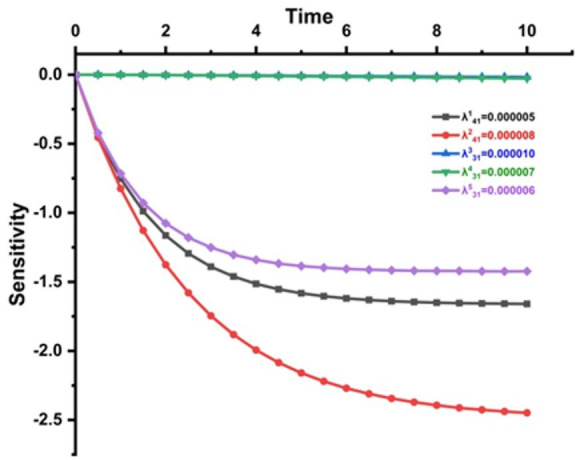
Sensitivity of DFIG for $\:\boldsymbol{K}=30$.

### Sensitivity analysis

Sensitivity analysis is essential in reliability theory for identifying which parameters, such as failure rates, degradation thresholds, or environmental factors, most critically influence system reliability. It validates model credibility by testing whether small input uncertainties lead to significant output variations, thereby assessing robustness. The sensitivity of the dynamic availability of the *r*^th^ component w.r.t. failure rate ($\:\lambda\:$) of the Doubly-Fed Induction Generator is expressed as:

12 $$S_{{\lambda _{{ij}} }}^{r} \left( t \right) = \frac{{\partial A\left( t \right)}}{{\partial \lambda _{{ij}}^{r} }}$$

**Fig. 31:**
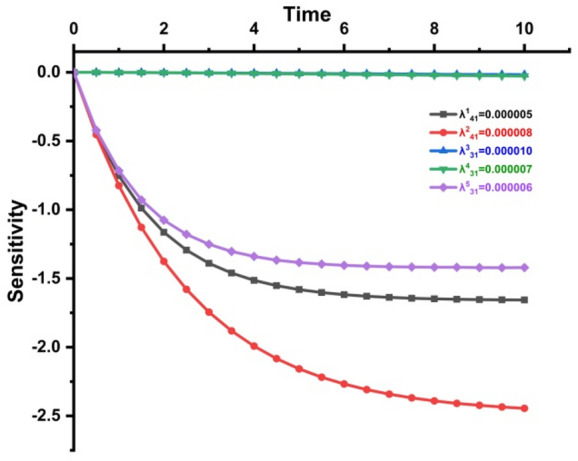
Sensitivity of DFIG for $\:\boldsymbol{K}=20$.

For *K* values of 40, 30, and 20, the sensitivity of each component within the DFIG system is calculated using Eq. (12) based on the post-maintenance failure rates. Here $\:{\lambda\:}_{ij}^{r}$ represents the failure rate of component * r*, transitioning from state * i* to state * j.* The resulting sensitivity values of each component for $\:K=\:$40, 30, and 20 are graphically presented in Figs. 29 and 30, and 31, respectively. These figures illustrate how variations in failure rates affect the availability of individual components, offering key insights into system performance and its vulnerability to specific failure modes.

For $\:K\:=\:40$ and 30, the sensitivity values for components 1, 2, and 5 increase steadily over time, with component 2 exhibiting the highest sensitivity (reaching − 2.44 at $\:t\:=\:10$), while components 3 and 4 show negligible sensitivity (on the order of 10⁻²). In contrast, at $\:K\:=\:20$, the sensitivity magnitudes increase dramatically for components 1, 2, and 5, particularly component 5, which becomes the most critical (-5.70 at $\:t\:=\:10$), indicating substantially higher system vulnerability to failure rate variations. However, an inverse trend is observed for components 3 and 4, where sensitivity decreases at $\:K\:=\:20$ compared to higher * K* values, suggesting a differential impact of the parameter * K* across failure modes. Overall, lower * K* values amplify the system’s responsiveness to changes in failure rates, especially for components associated with $\:{\lambda\:}_{41}$ and the fifth component’s $\:{\lambda\:}_{31}$. These results highlight the importance of prioritizing maintenance strategies based on both component identity and the operational $\:K\:$regime.

## Conclusion

In this paper, a novel model, based on $\:{L}_{z}$*-* transform, has been developed to incorporate the simultaneous external extreme shock and internal degradation. The motivation behind the study lies in the fact that most of the systems working in external environment experience some external shock over it. In addition to that, the system continuously undergoes internal degradation due to aging. The proposed model is then applied on the Doubly-Fed Induction Generator (DFIG) as a case study and the dynamic reliability measures have been obtained with the help of $\:{L}_{z}$*-* transform. The results indicate that both availability and reliability deteriorate drastically over time, asymptotically approaching zero, due to the combined effects of internal degradation and external shocks on DFIG’s components. Moreover, the reliability decreases largely as compared to availability as time continues due to repair activities being carried for the availability.

In addition to the proposed model, a new maintenance strategy has been also proposed based on dummy states. The results show that both the reliability and availability increases to near-perfect availability and reliability (approximately 1.0) for most of the cases. These show the positive impact of maintenance on system’s performance. Also, the system behavior with respect to varying time and shock with constant performance is also obtained for DFIG system both with and without maintenance. The comparative analysis reveals that the implemented maintenance strategy is critically important, fundamentally transforming system resilience against shock-induced failure. It completely negates the time-dependent degradation observed in the non-maintained system, enabling a robust system (maximum bearable shock = 20) to sustain near-perfect availability (~ 1.00) indefinitely instead of decaying to a mere 0.00657. For vulnerable systems (maximum bearable shock = 15), maintenance provides a monumental improvement of five to six orders of magnitude. Crucially, the persistent invariance of availability to redundancy $\:\left(K\right)$ confirms that these dramatic benefits are solely attributable to the maintenance protocol, not additional components. This proves that maintenance is the paramount strategy for mitigating time-based wear-out and is indispensable for achieving high, sustainable availability in shock-prone environments.

## Data Availability

The datasets used and/ or analysed during the current study available from the corresponding author on reasonable request.

## Abbreviations

$\:{p}_{ij}$: Probability of the component i in state j
$\:{g}_{ij}$: Performance level of component i which is in state j
$\:{\lambda\:}_{ij}$: Transition (failure) rate of component from state i to state j (i > j)
$\:{\mu\:}_{ji}$: Transition (repair) rate of component from state j to state i (i > j)
State* M*: The state of the complete performance of the component
State* 1*: The state of the failure of the component
$\:\delta\:$: Intensity of extreme external shock
$\:{\delta\:}_{j}$: Maximum shock tolerance threshold of the any component operating in state* j*
$\:R\left(t\right)$: Dynamic reliability of system at any instant* t*
$\:A\left(t\right)$: Dynamic availability of system at any instant* t*
$\:E\left(t\right)$: Mean expected performance of the system at time* t*
$$\:{c}_{r}$$: Revenue cost
$$\:{c}_{s}$$: Service cost
$\:{S}_{{\lambda\:}_{ij}}^{r}\left(t\right)$: Sensitivity of r^th^ component with respect to failure rate
$\:{E}_{p}\left(t\right)$: Expected profit of the system at time* t*
